# Supplementary material for: Proton signaling links epithelial sensing to neural control of host defense in C. elegans
Source: Nat Commun. 2026 Mar 27;17:4493. doi: 10.1038/s41467-026-71088-6 (PMC13187020; doi:10.1038/s41467-026-71088-6)

Supplementary Information

Proton signaling links epithelial sensing to neural control of host defense in *C. elegans*

Ying Lei, Xu Zhan, Chao Chen, Yuxin Liu, Ying Wang, and Ping Liu

Supplementary Figures 1-11 and Supplementary Tables 1 and 2

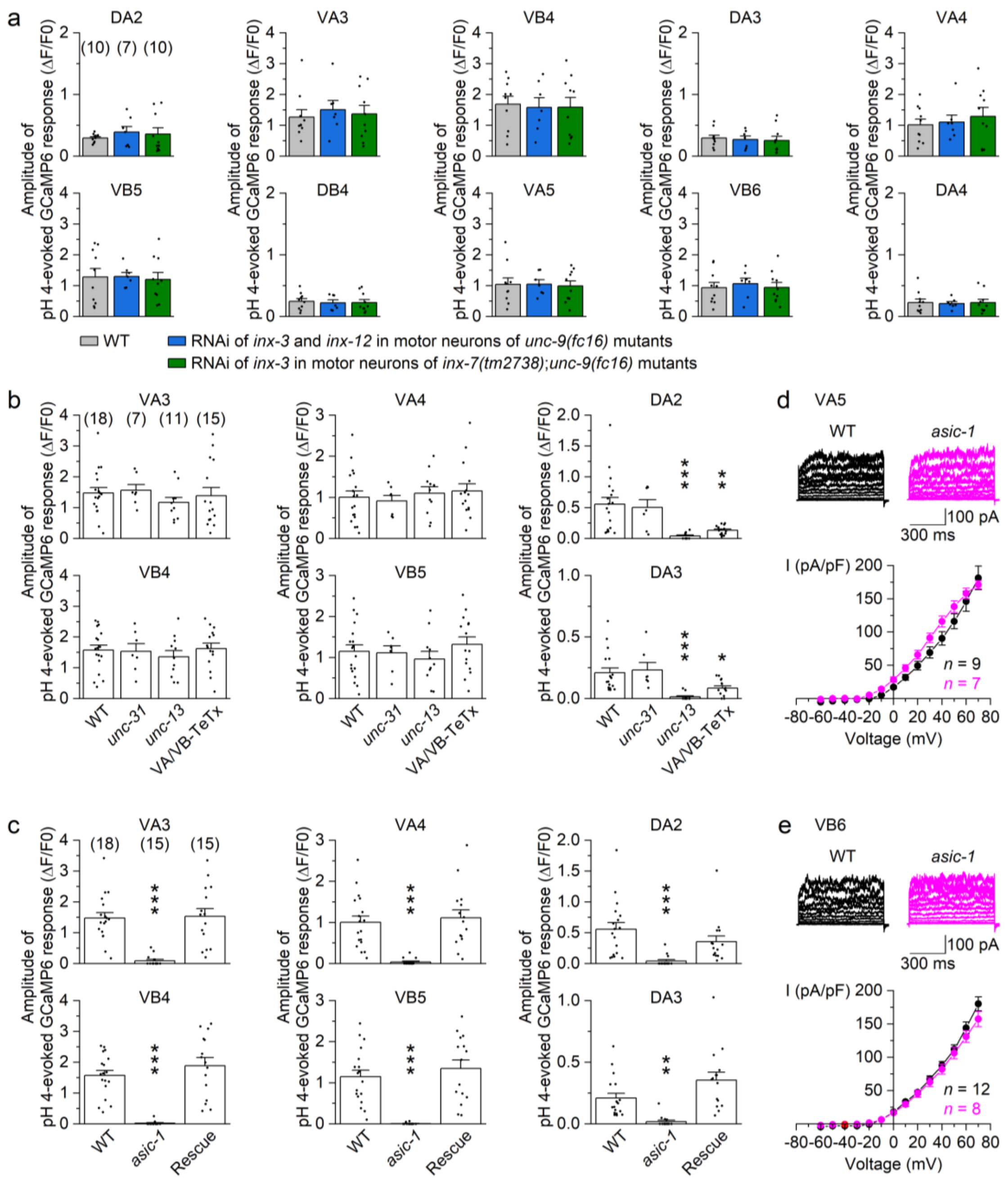

Supplementary Fig. 1 ASIC-1 is required for the acid sensitivity of VA and VB motor neurons. a-c, Comparisons of pH 4-evoked GCaMP6 amplitude in A- and B-type motor neurons of the indicated genotypes. WT, wild type. RNAi of *inx-3* and *inx-12* was done in cholinergic VNC motor neurons using *Punc-17Δ1*. VA/VB-TeTx, transgenic worms expressing tetanus toxin (TeTx) in VA and VB using *Pdel-1*. Rescue was done by expressing wild-type *asic-1* in VA and VB using *Pdel-1*. p = 0.68415, 0.80547, 0.81517, 0.95453, 0.97188, 0.96935, 0.96554, 0.86364, 0.96532, 0.67721, 0.99788, 0.96381, 0.94244, 0.94767, 0.99911, 0.97581, 0.85246, 0.99879, 0.97201,

and 0.9997 (**a**), 0.9949, 0.73923, 0.9877, 0.98682, 0.97599, 0.88833, 0.98045, 0.0004, 0.0014, 0.99911, 0.83361, 0.99696, 0.99919, 0.87946, 0.87025, 0.97069, 0.0005, and 0.0229 (**b**), and < 0.0001, 0.97236, < 0.0001, 0.85422, 0.0004, 0.23891, < 0.0001, 0.408, < 0.0001, 0.61728, 0.00745, and 0.0477 (**c**). **d,e,** Whole-cell currents of VA5 and VB6 (representatives of VA and VB, respectively) in response to membrane voltage steps ranging from -60 mV to +70 mV in 10-mV intervals, starting from a holding voltage of -60 mV. Left, sample traces. Right, current-voltage relationships of WT and *asic-1(ok415)* mutants. $p = 0.27729$ (**d**) and 0.57668 (**e**). *$p < 0.05$, **$p < 0.01$, and ***$p < 0.001$ (one-way ANOVA with Tukey's post hoc test for **a-c**; two-way repeated-measures ANOVA with Sidak's multiple-comparisons correction for **d,e**). Brackets contain the number of animals tested (*n*). Data are shown as means ± SEM. Source data are provided as a Source Data file.

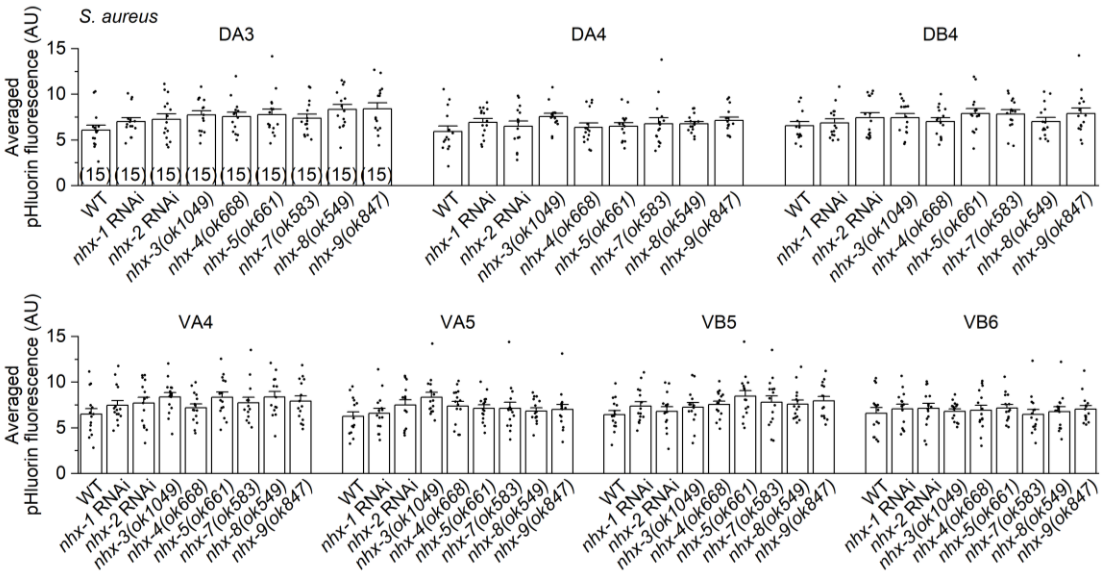

Supplementary Fig. 2 IEC-specific RNAi or mutations in other Na$^+$/H$^+$ exchanger (NHE) genes do not affect pHluorin fluorescence in motor neurons during *S. aureus* infection. Comparisons of averaged pHluorin fluorescence among the indicated genotypes. WT, wild type. IEC-specific RNAi was done using *Pges-1*. AU, arbitrary units. p = 0.94592, 0.82665, 0.43534, 0.57934, 0.40838, 0.75184, 0.07967, 0.06604, 0.85396, 0.99532, 0.26818, 0.99903, 0.99443, 0.94669, 0.94602, 0.67498, 0.99995, 0.9325, 0.94309, 0.99894, 0.59962, 0.6497, 0.999, 0.61381, 0.94804, 0.82724, 0.3026, 0.99362, 0.31663, 0.8271, 0.32566, 0.68704, 0.99993, 0.7359, 0.11182, 0.82905, 0.9542, 0.95325, 0.99631, 0.98065, 0.94136, 0.99989, 0.97456, 0.85617, 0.14515, 0.64788, 0.83622, 0.51788, 0.99878, 0.99774, 1, 0.99995, 0.99642, 1, 1, and 0.99941. No significant differences were observed (one-way ANOVA with Tukey's post hoc test). Brackets contain the number of animals tested (n). Data are shown as means ± SEM. Source data are provided as a Source Data file.

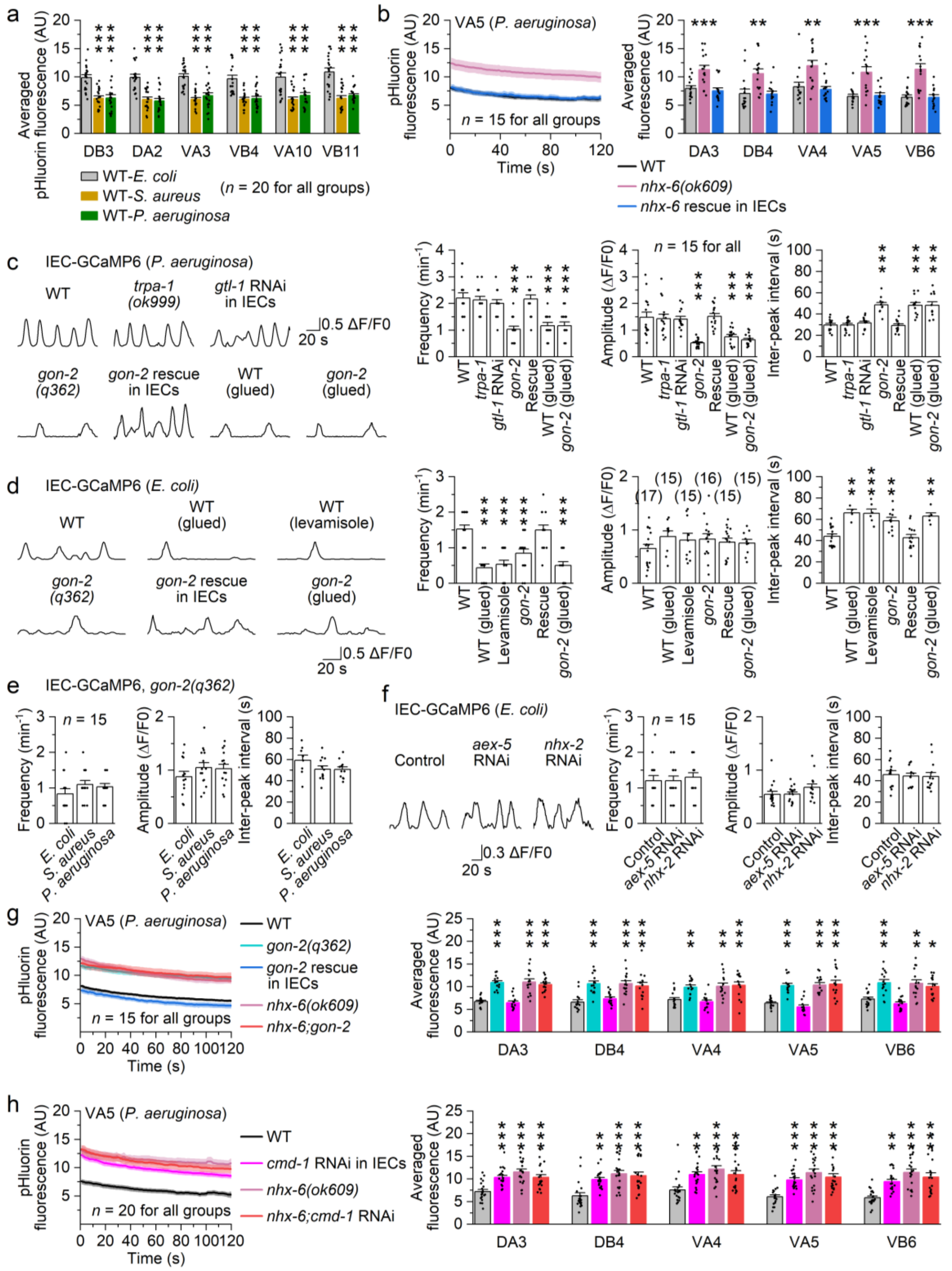

Supplementary Fig. 3 ***P. aeruginosa*** **infection induces IEC proton secretion via GON-2-CMD-1-NHX-6 signaling. a,** Averaged pHluorin fluorescence measured at multiple distal motor neurons distributed along the anterior and posterior VNC in wild-type (WT) worms fed on the indicated bacteria. AU, arbitrary units. p = < 0.0001, < 0.0001, < 0.0001, < 0.0001, < 0.0001, < 0.0001, < 0.0001, < 0.0001, < 0.0001, 0.0007, < 0.0001, and < 0.0001. **b,** pHluorin fluorescence in motor neurons of WT, *nhx-*

6(ok609), and *nhx-6* rescue worms fed on *P. aeruginosa*. Left, fluorescence of VA5 as representatives. Right, comparisons. Rescue was done by expressing wild-type *nhx-6* in IECs using *Pges-1*. p = 0.0009, 0.88502, 0.00373, 0.99819, 0.0023, 0.90063, < 0.0001, 0.9798, < 0.0001, and 0.99988. **c,d,f,** Spontaneous GCaMP6 signals in IECs of worms with the indicated genotypes and conditions fed on *P. aeruginosa* (**c**) or *E. coli* (**d,f**). Left, sample traces. Right, comparisons of the frequency, amplitude, and inter-peak interval of spontaneous Ca^{2+} transients. IEC-specific RNAi of *gtl-1* was done using *Pges-1*. IEC-specific RNAi of *aex-5* and *nhx-2* was done by feeding *C. elegans* strain VP303 with *E. coli* HT115 carrying L4440 plasmids containing gene fragments or empty vector as control. p = 0.99989, 0.9546, < 0.0001, 1, < 0.0001, < 0.0001, 0.9999, 0.99953, < 0.0001, 1, 0.0007, < 0.0001, 1, 0.99021, < 0.0001, 0.99999, < 0.0001, and < 0.0001 (**c**), < 0.0001, < 0.0001, 0.0006, 0.99997, < 0.0001, 0.57793, 0.83427, 0.67783, 0.91132, 0.96904, 0.00227, 0.0003, 0.00555, 0.9993, and 0.0046 (**d**), and 1, 0.86258, 0.99968, 0.20712, 0.96911, and 0.92955 (**f**). **e,** Comparisons of the frequency, amplitude, and inter-peak interval of spontaneous Ca^{2+} transients in IECs of *gon-2(q362)* mutants fed on the indicated bacteria. p = 0.25365, 0.45645, 0.40444, 0.50079, 0.22195, and 0.21986. **g,h,** pHluorin fluorescence in motor neurons of the indicated genotypes fed on *P. aeruginosa*. Left, fluorescence of VA5 as representatives. Right, comparisons. Rescue was done by expressing wild-type *gon-2* in IECs using *Pges-1*. IEC-specific RNAi of *cmd-1* was done using *Pges-1*. p = < 0.0001, 0.99439, < 0.0001, < 0.0001, < 0.0001, 0.87811, < 0.0001, 0.0005, 0.00413, 0.96684, 0.00183, 0.0007, < 0.0001, 0.77351, < 0.0001, < 0.0001, 0.0005, 0.76469, 0.00105, and 0.01288 (**g**), and 0.0006, < 0.0001, 0.0006, 0.0012, < 0.0001, < 0.0001, 0.00583, < 0.0001, 0.00535, 0.0002, < 0.0001, < 0.0001, 0.00151, < 0.0001, and < 0.0001 (**h**). *p < 0.05, **p < 0.01, and ***p < 0.001 (one-way ANOVA with Tukey's post hoc test). Brackets contain the number of animals tested (n). Data are shown as means ± SEM. Source data are provided as a Source Data file.

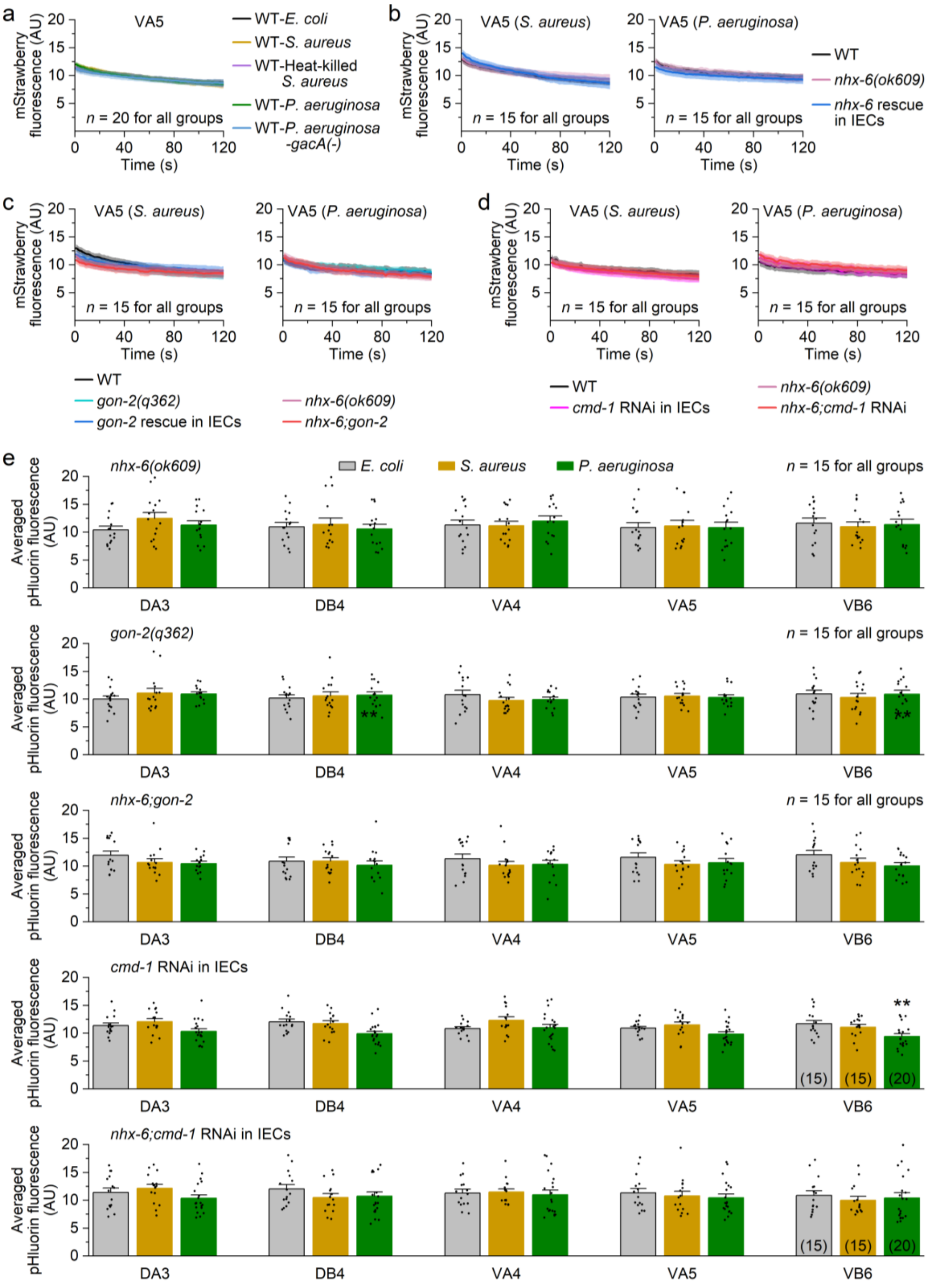

Supplementary Fig. 4 mStrawberry expression control and loss of *nhx-6*, *gon-2*, or *cmd-1* abolishes pathogen-induced changes in pHluorin fluorescence in VNC motor neurons. a-d, mStrawberry fluorescence in motor neurons of pHluorin transgenic worms with the indicated genotypes fed on *E. coli* or pathogenic bacteria. mStrawberry was co-expressed with pHluorin under *Punc-17Δ1* and is shown as a pH-

insensitive expression control. Fluorescence of VA5 was shown as representatives. Solid lines and shaded regions indicate the mean and SEM, respectively. AU, arbitrary units. **e,** Comparisons of averaged pHluorin fluorescence in motor neurons of the indicated genotypes fed on *E. coli* or pathogenic bacteria. IEC-specific RNAi of *cmd-1* was done using *Pges-1*. Data for worms fed on pathogenic bacteria are reused from Fig. 2c,f,g and Supplementary Fig. 3b,g,h. p = 0.19653, 0.74882, 0.93383, 0.95106, 0.99472, 0.81745, 0.97158, 0.99963, 0.87444, 0.9829, 0.44842, 0.54351, 0.90095, 0.82948, 0.4693, 0.60009, 0.93404, 0.99898, 0.79809, 0.99989, 0.3175, 0.21067, 0.99963, 0.75337, 0.49809, 0.60985, 0.47366, 0.62999, 0.36655, 0.12572, 0.60291, 0.32125, 0.90944, 0.00629, 0.17653, 0.97965, 0.66655, 0.17872, 0.73934, 0.00993, 0.76385, 0.5157, 0.36966, 0.4485, 0.98334, 0.95837, 0.88531, 0.69186, 0.78397, and 0.92899. **$p < 0.01$ (one-way ANOVA with Tukey's post hoc test). Brackets contain the number of animals tested (n). Data are shown as means ± SEM. Source data are provided as a Source Data file.

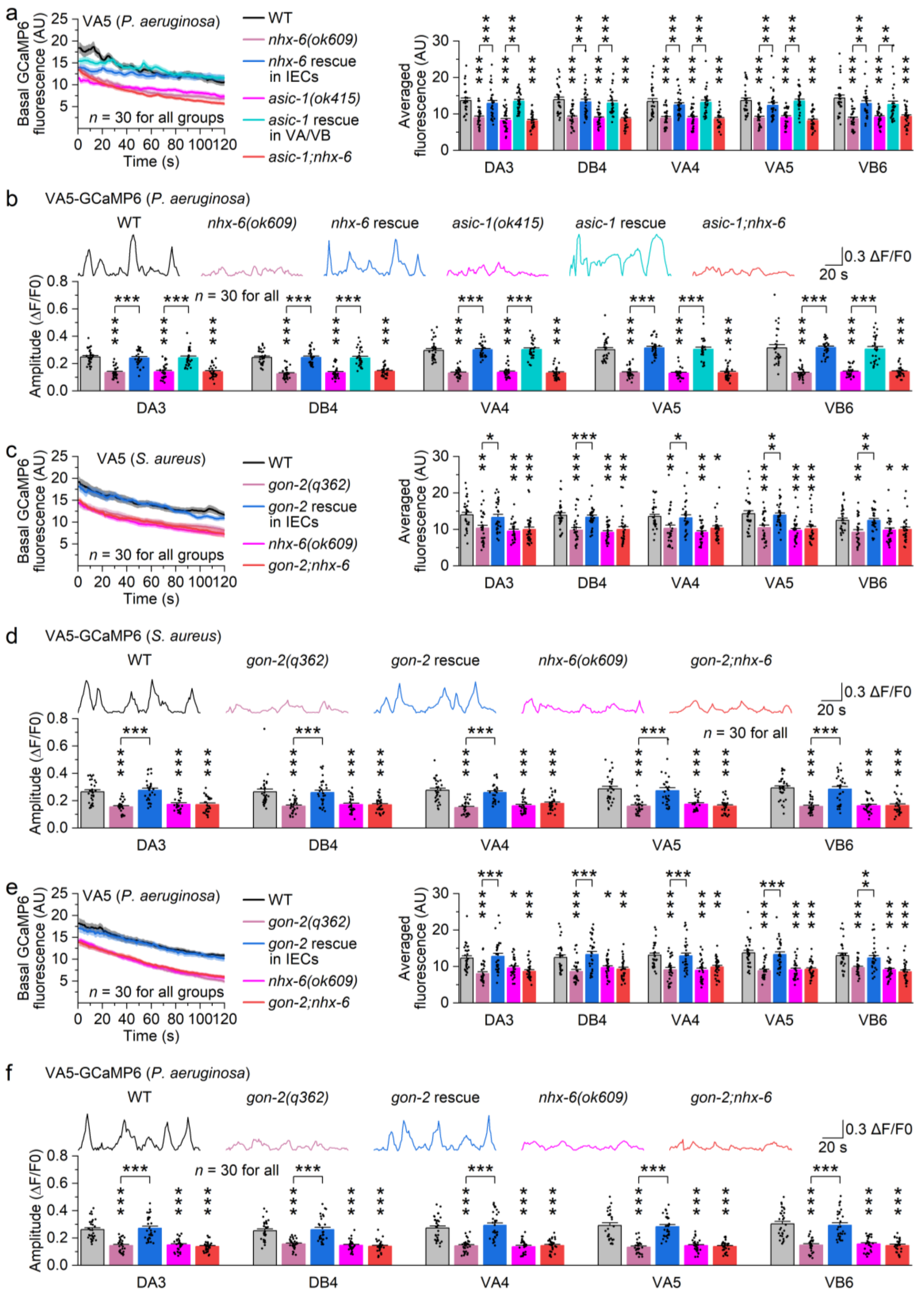

Supplementary Fig. 5 ***P. aeruginosa*** **infection activates motor neurons through NHX-6-H$^+$-ASIC-1 signaling between the intestine and motor neurons. a,c,e,** Basal GCaMP6 fluorescence in motor neurons of the indicated genotypes fed on *P. aeruginosa* or *S. aureus*. Left, fluorescence of VA5 as representatives. Solid lines and

shaded regions indicate the mean and SEM, respectively. Right, comparisons of averaged basal fluorescence. AU, arbitrary units. WT, wild type. Rescues were done by expressing wild-type *nhx-6* or *gon-2* in IECs using *Pges-1* and *asic-1* in VA/VB using *Pdel-1*, respectively. *p* = < 0.0001, 0.92699, < 0.0001, < 0.0001, 0.99974, < 0.0001, < 0.0001, < 0.0001, 0.97021, < 0.0001, < 0.0001, 0.85551, < 0.0001, < 0.0001, < 0.0001, 0.82619, 0.0001, < 0.0001, 0.99946, < 0.0001, < 0.0001, < 0.0001, 0.57732, 0.0002, < 0.0001, 0.99999, < 0.0001, < 0.0001, < 0.0001, 0.59429, < 0.0001, < 0.0001, 0.38174, 0.00203, and < 0.0001 (**a**), 0.00244, 0.96339, 0.02111, 0.0001, 0.0006, < 0.0001, 0.97842, 0.0008, < 0.0001, 0.0002, 0.00992, 0.99694, 0.02799, 0.0001, 0.01126, 0.0006, 0.99225, 0.00308, < 0.0001, 0.0001, 0.00219, 1, 0.00211, 0.01599, and 0.03028 (**c**), and < 0.0001, 0.96854, < 0.0001, 0.01077, 0.0002, 0.0002, 0.89648, < 0.0001, 0.02003, 0.00464, < 0.0001, 0.99934, 0.0002, < 0.0001, 0.00182, < 0.0001, 0.98169, < 0.0001, < 0.0001, < 0.0001, 0.0002, 0.91143, 0.00512, < 0.0001, and < 0.0001 (**e**). **b,d,f,** Spontaneous GCaMP6 signals in motor neurons of the indicated genotypes fed on *P. aeruginosa* or *S. aureus*. Top, sample traces of VA5 as representatives. Bottom, comparisons of the amplitude of spontaneous Ca^{2+} transients. *p* = 0, 0.99161, 0, 0, 0.99916, 0, 0, 0, 1, < 0.0001, 0, 0.99996, 0, 0, 0, 0.99969, 0, 0, 0.99774, 0, 0, 0, 0.95523, 0, 0, 0.99998, 0, 0, < 0.0001, 0.99999, < 0.0001, 0, 0.99853, 0, and 0 (**b**), < 0.0001, 0.96766, < 0.0001, < 0.0001, < 0.0001, < 0.0001, 0.99828, < 0.0001, < 0.0001, < 0.0001, < 0.0001, 0.84875, < 0.0001, < 0.0001, < 0.0001, < 0.0001, 0.9681, < 0.0001, < 0.0001, < 0.0001, < 0.0001, 0.98954, < 0.0001, < 0.0001, and < 0.0001 (**d**), and < 0.0001, 0.96883, 0, < 0.0001, 0, < 0.0001, 0.97989, < 0.0001, < 0.0001, < 0.0001, < 0.0001, 0.79824, 0, < 0.0001, < 0.0001, 0, 0.98891, 0, 0, 0, 0, 0.98138, < 0.0001, < 0.0001, and 0 (**f**). *p < 0.05, **p < 0.01, and ***p < 0.001 (one-way ANOVA with Tukey's post hoc test). *n* represents the number of animals tested. Data are shown as means ± SEM. Source data are provided as a Source Data file.

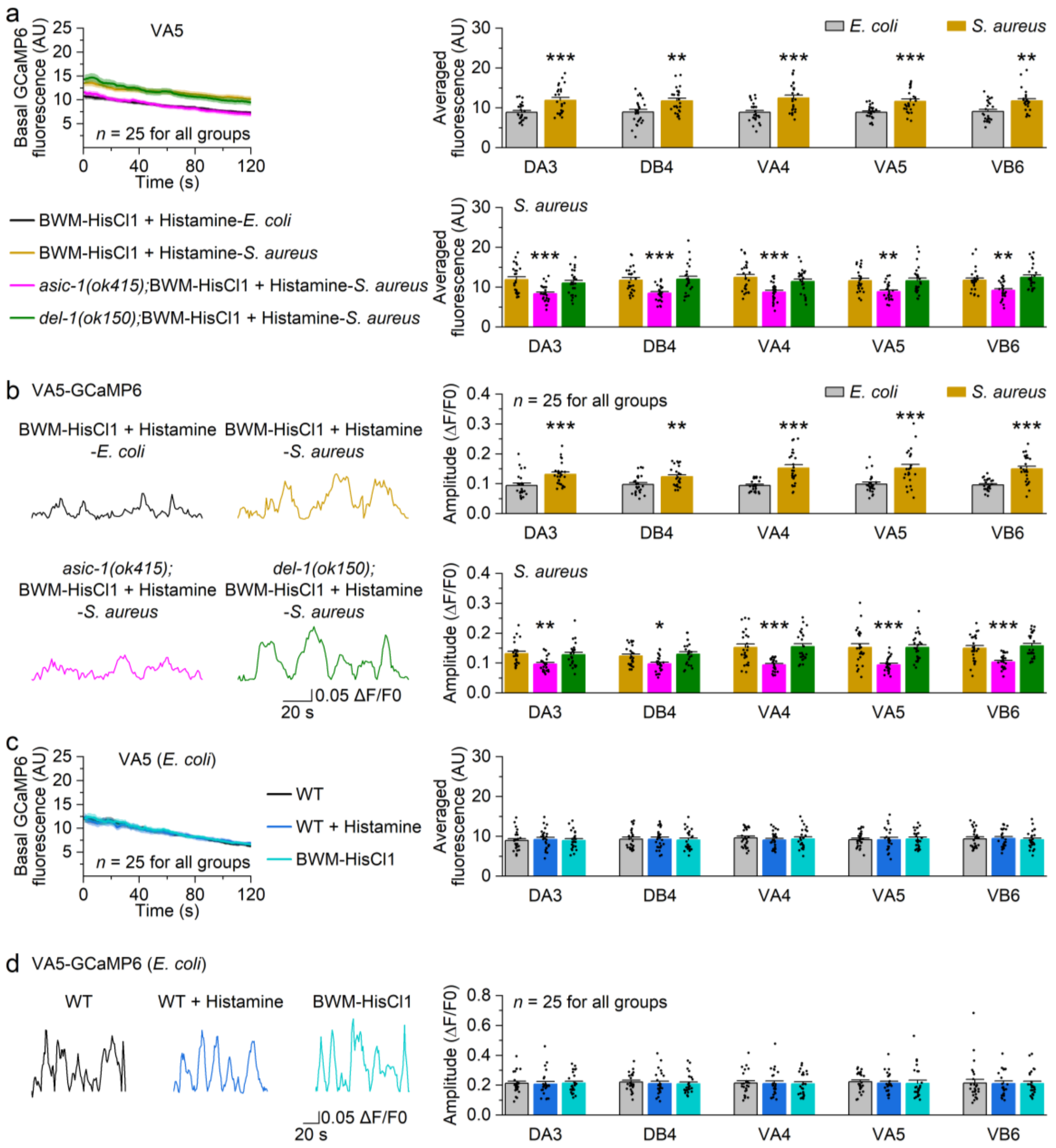

Supplementary Fig. 6 ASIC-1 but not DEL-1 is required for the intestinal pathogen-induced activation of motor neurons. a,c, Basal GCaMP6 fluorescence in motor neurons of worms with the indicated genotypes and conditions raised in *E. coli* or *S. aureus* liquid cultures. Left, fluorescence of VA5 as representatives. Solid lines and shaded regions indicate the mean and SEM, respectively. Right, comparisons of averaged basal fluorescence. AU, arbitrary units. WT, wild type. To paralyze worms, a transgenic strain expressing HisCl1 in body-wall muscles (BWM-HisCl1) was used, and 50 mM histamine was added to the liquid cultures to ensure bacterial ingestion despite immobility. p = 0.0005, 0.00234, 0.0002, 0.0001, 0.00101, 0.0002, 0.5608, 0.0006, 0.96101, 0.0001, 0.44313, 0.00283, 0.99909, 0.00378, and

0.59709 (**a**), and 0.88893, 0.99749, 0.99966, 0.93097, 0.7284, 0.94091, 0.99998, 0.95197, 0.98979, and 0.93077 (**c**). **b,d,** Spontaneous GCaMP6 signals in motor neurons of worms with the indicated genotypes and conditions raised in *E. coli* or *S. aureus* liquid cultures. Left, sample traces of VA5 as representatives. Right, comparisons of the amplitude of spontaneous Ca^{2+} transients. p = 0.00096, 0.00447, < 0.0001, 0.0002, < 0.0001, 0.00151, 0.91166, 0.01417, 0.75107, < 0.0001, 0.98118, < 0.0001, 0.99952, 0.0002, and 0.75853 (**b**), and 0.97933, 0.99635, 0.85759, 0.78399, 0.99498, 0.97471, 0.91864, 0.89359, 0.9946, and 0.99391 (**d**). *p < 0.05, **p < 0.01, and ***p < 0.001 (one-way ANOVA with Tukey's post hoc test). *n* represents the number of animals tested. Data are shown as means ± SEM. Source data are provided as a Source Data file.

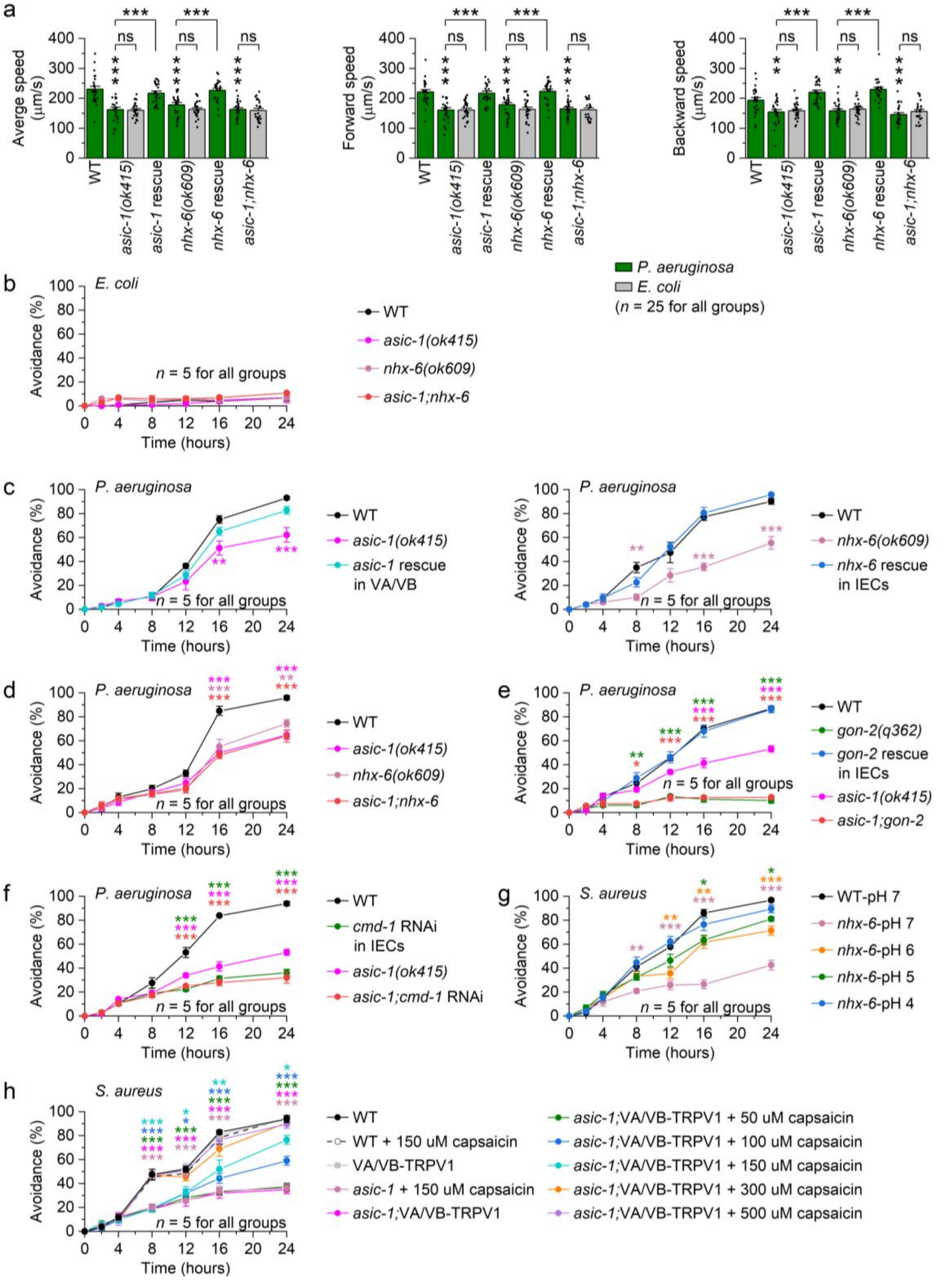

Supplementary Fig. 7 NHX-6-H^{+}-ASIC-1 signaling enhances pathogen avoidance. **a,** Comparisons of average, forward, and backward speed among the indicated genotypes fed on *P. aeruginosa*. WT, wild type. Rescues were done by expressing wild-type *nhx-6* in IECs using *Pges-1* and *asic-1* in VA/VB using *Pdel-1*, respectively. Data for worms fed on *E. coli* are reused from Fig. 4b. p = < 0.0001, 1, 0.92851, <

0.0001, < 0.0001, 0.86336, 0.99999, < 0.0001, < 0.0001, 0.99999, < 0.0001, 1, 0.99998, < 0.0001, 0.0007, 0.82433, 1, 0.0002, < 0.0001, 0.99999, 0.00196, 0.99999, 0.12109, < 0.0001, 0.00753, 0.99953, 0.00677, < 0.0001, < 0.0001, and 0.9826. **b-f,** Pathogen avoidance of the indicated genotypes on the partial lawn of *E. coli* (**b**) or *P. aeruginosa* (**c-f**). Rescue was done by expressing wild-type *gon-2* in IECs using *Pges-1*. IEC-specific RNAi of *cmd-1* was done using *Pges-1*. p = 0.9855, 0.25063, 0.05878 (**b**), 0.98096, 0.99999, 0.99999, 0.21414, 0.00598, 0.00037, 0.99972, 0.66365, 0.99073, 0.62724, 0.33187, 0.2369, 1, 0.76099, 0.00139, 0.14075, < 0.0001, < 0.0001, 0.99995, 0.9996, 0.10147, 0.93578, 0.90247, and 0.6182 (**c**), 1, 0.84578, 0.95711, 0.5104, < 0.0001, 0.00011, 0.99993, 0.99642, 0.74675, 0.07185, 0.0005, 0.00444, 0.99731, 0.99988, 0.85606, 0.11495, < 0.0001, < 0.0001 (**d**), 0.99999, 0.24035, 0.00512, < 0.0001, < 0.0001, < 0.0001, 1, 0.92722, 0.98819, 1, 0.99998, 1, 0.9988, 1, 0.94502, 0.06687, < 0.0001, < 0.0001, 0.57301, 0.54957, 0.01064, < 0.0001, < 0.0001, < 0.0001 (**e**), and 1, 1, 0.37993, < 0.0001, < 0.0001, < 0.0001, 0.99593, 0.38522, 0.38275, 0.00035, < 0.0001, < 0.0001, 1, 1, 0.18288, < 0.0001, < 0.0001, < 0.0001 (**f**). **g,** Pathogen avoidance of WT and *nhx-6(ok609)* mutants on the partial lawn of *S. aureus* at the indicated pH. p = 0.99789, 0.99539, 0.0023, 0.00015, < 0.0001, < 0.0001, 0.82957, 1, 0.60925, 0.00847, 0.00737, < 0.0001, 0.81705, 0.99826, 0.50599, 0.448848, 0.01475, 0.01606, 1, 1, 0.99695, 0.9974, 0.75918, and 0.68001. **h,** Effects of capsaicin on pathogen avoidance of the indicated genotypes. VA/VB-TRPV1, transgenic worms expressing TRPV1 in VA and VB using *Pdel-1*. Capsaicin was added to the worm culture plates. p = 1, 1, 1, 1, 1, 1, 1, 1, < 0.0001, 0.00018, < 0.0001, < 0.0001, < 0.0001, 0.00025, < 0.0001, < 0.0001, < 0.0001, 0.00091, < 0.0001, < 0.0001, < 0.0001, 0.01087, < 0.0001, < 0.0001, < 0.0001, 0.01446, 0.00145, 0.01762, 1, 1, 0.84357, 1, 1, 1, 1, and 1. *p < 0.05, **p < 0.01, and ***p < 0.001 (one-way ANOVA with Tukey's post hoc test for **a**; two-way repeated-measures ANOVA with Sidak's multiple-comparisons correction for **b-h**). ns, no significance. n represents the number of animals tested (**a**) or independent assays (**b-h**). Data are shown as means ± SEM. Source data are provided as a Source Data file.

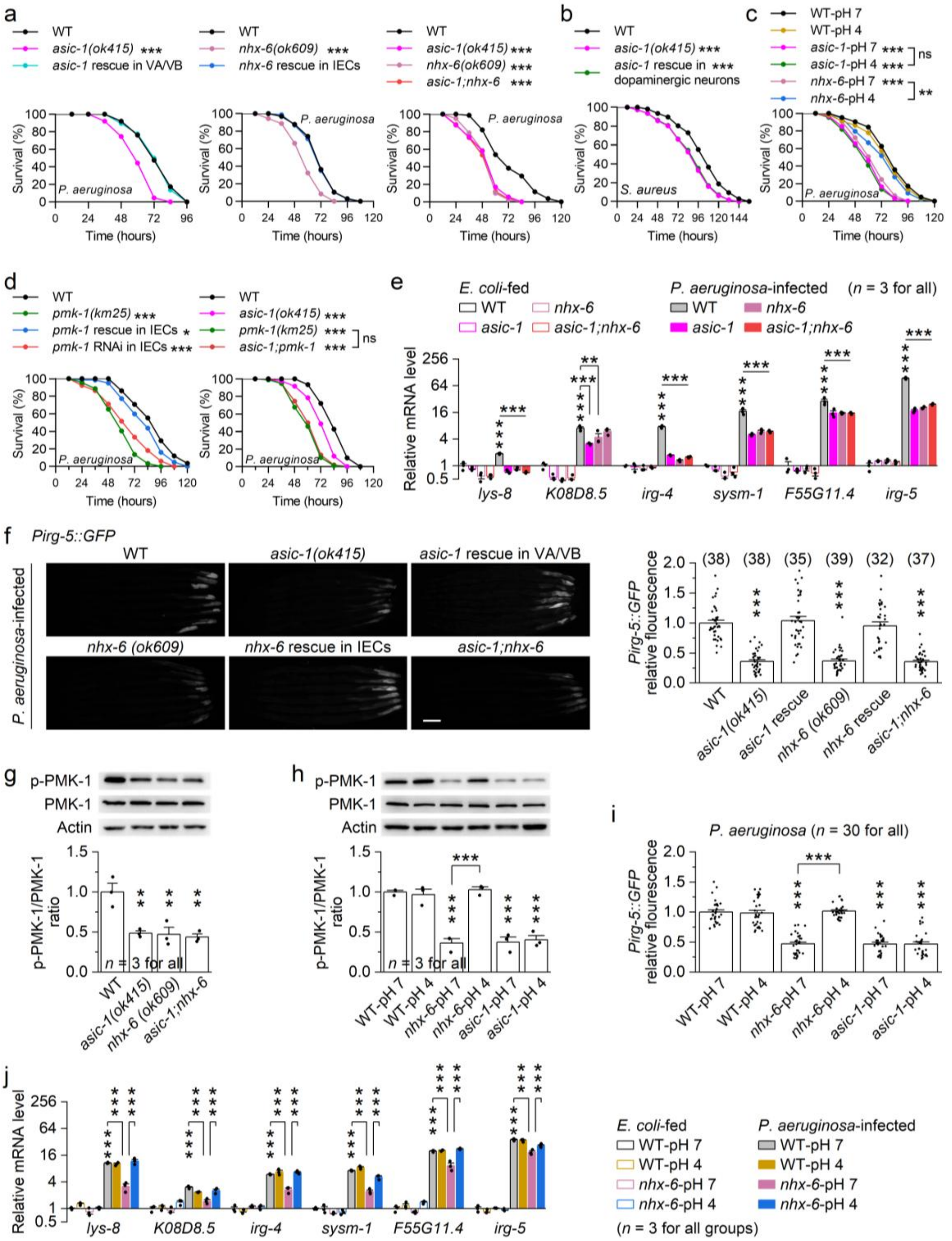

Supplementary Fig. 8 NHX-6-H$^+$-ASIC-1 signaling promotes intestinal innate immunity through the PMK-1/p38 MAPK pathway. a,d, Percent survival of the indicated genotypes on the full lawn of *P. aeruginosa*. WT, wild type. Rescues were done by expressing wild-type *asic-1* in VA/VB using *Pdel-1*, *nhx-6* in IECs using *Pges-1*, and *pmk-1* in IECs using *Pges-1*, respectively. IEC-specific RNAi of *pmk-1* was done using *Pges-1*. p = < 0.0001, 0.9765, < 0.0001, 0.7995, < 0.0001, < 0.0001, and <

0.0001 (**a**), and < 0.0001, 0.0393, < 0.0001, < 0.0001, < 0.0001, < 0.0001, and 0.3753 (**d**). **b,** Expression of wild-type *asic-1* in dopaminergic neurons using *Pdat-1* did not rescue the reduced survival of *asic-1(ok415)* mutants against *S. aureus* infection. *p* = < 0.0001 and 0.0002. **c,** Percent survival of WT, *asic-1(ok415)*, and *nhx-6(ok609)* worms on the full lawn of *P. aeruginosa* at pH 7 or 4. *p* = 0.4286, < 0.0001, < 0.0001, 0.5501, < 0.0001, 0.084, and 0.005. **e,** qRT-PCR analyses of the expression levels of six PMK-1/p38-dependent immune genes in the indicated genotypes fed on *E. coli* or *P. aeruginosa*. *p* = 0.72251, 0.00227, 0.0025, < 0.0001, < 0.0001, < 0.0001, < 0.0001, 0.99348, 0.98527, 0.99176, < 0.0001, 0.0001, 0.00884, 0.64825, 0.99997, 0.9951, 0.99985, 0, 0, 0, 0, 1, 0.99948, 0.99996, 0, < 0.0001, < 0.0001, < 0.0001, 1, 1, 1, < 0.0001, < 0.0001, < 0.0001, < 0.0001, 1, 1, 1, 0, 0, 0, and 0. **f,** Expression of *Pirg-5::GFP* in the indicated genotypes fed on *P. aeruginosa*. Shown are representative images and comparisons of relative fluorescence normalized to wild-type controls. Scale bars, 100 μm. *p* = 0, 0.98796, 0, 0.98209, and 0. **g,** Representative immunoblots and quantification of the ratio of phosphorylated PMK-1 (p-PMK-1) to total PMK-1 in the indicated genotypes fed on *P. aeruginosa*. β-actin served as a loading control. *p* = 0.005, 0.00414, and 0.00286. **h,** Representative immunoblots and quantification of the ratio of p-PMK-1 to total PMK-1 in WT, *asic-1(ok415)*, and *nhx-6(ok609)* worms fed on *P. aeruginosa* at pH 7 or 4. *p* = 0.9977, < 0.0001, 0.99864, < 0.0001, < 0.0001, < 0.0001, and 0.9987. **i,** Comparisons of *Pirg-5::GFP* relative fluorescence in WT, *asic-1(ok415)*, and *nhx-6(ok609)* worms fed on *P. aeruginosa* at pH 7 or 4. *p* = 0.99963, 0, 0.99965, 0, 0, 0, and 1. **j,** qRT-PCR analyses of the expression levels of six PMK-1/p38-dependent immune genes in WT and *nhx-6(ok609)* worms fed on *E. coli* or *P. aeruginosa* at pH 7 or 4. *p* = 0.99978, 1, 1, < 0.0001, 0.98378, < 0.0001, 0.68271, < 0.0001, 0.99963, 0.99903, 0.26079, < 0.0001, 0.01114, < 0.0001, 0.1317, < 0.0001, 0.99998, 0.99996, 1, < 0.0001, 0.37251, < 0.0001, 0.5246, < 0.0001, 1, 0.99387, 0.9982, 0, 0.0057, < 0.0001, < 0.0001, < 0.0001, 1, 1, 0.99955, 0, 0.99421, < 0.0001, 0.13853, < 0.0001, 1, 1, 1, 0, 0.92058, < 0.0001, < 0.0001, and 0.0007. **p* < 0.05, ***p* < 0.01, and ****p* < 0.001 (log-rank (Kaplan-Meier) test for **a-d**; one-way ANOVA with Tukey's post hoc test for **e-j**). ns, not significant. Brackets contain the number of

animals tested (*n*). Data are shown as means ± SEM. Source data are provided as a Source Data file.

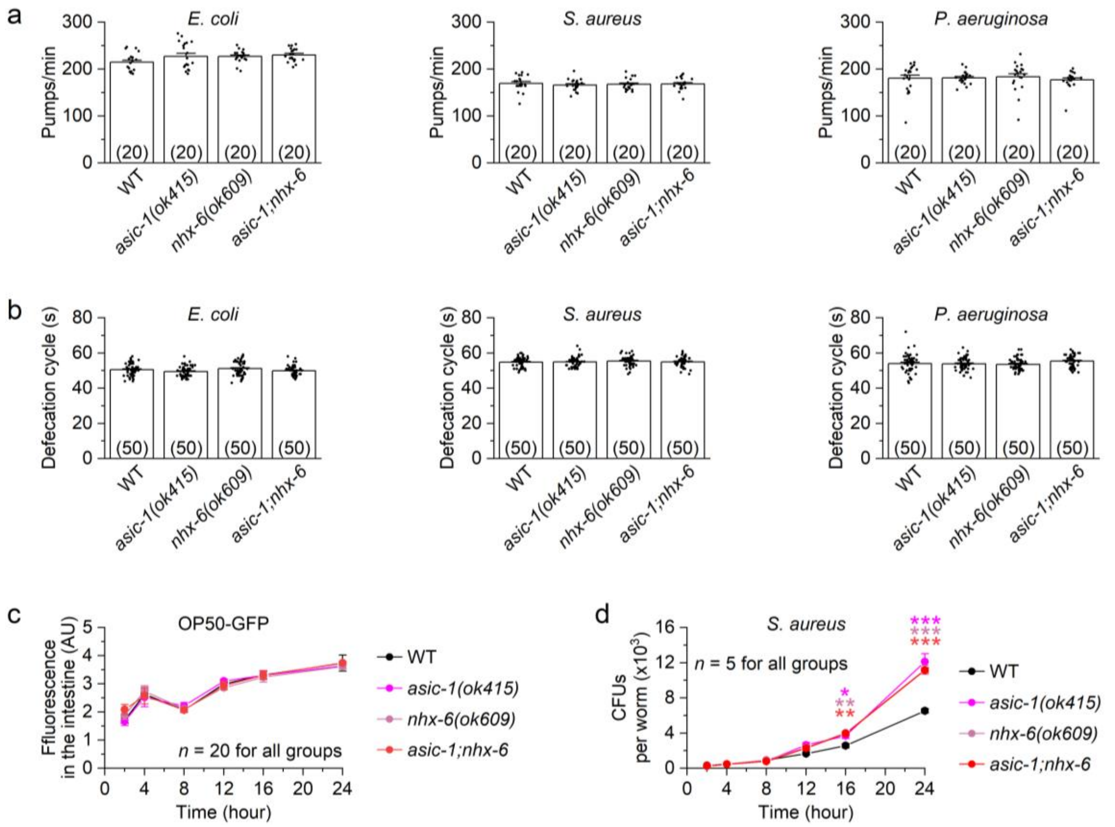

Supplementary Fig. 9 Intestinal accumulation of *S. aureus* is increased in *asic-1(ok415)* and *nhx-6(ok609)* mutants. a,b, Pharyngeal pumping rates (**a**) and defecation cycle durations (**b**) of wild-type (WT), *asic-1(ok415)*, *nhx-6(ok609)*, and *asic-1;nhx-6* worms fed on the indicated bacteria. Pharyngeal pumping rate was quantified as the number of terminal bulb contractions per minute and serves as a measure of feeding activity. p = 0.19264, 0.20466, 0.07699, 0.82552, 0.95894, 0.98923, 0.99921, 0.97779, and 0.97106 (**a**), and 0.40724, 0.83014, 0.81532, 0.99794, 0.65998, 0.99442, 0.99607, 0.95411, and 0.27217 (**b**). **c,** Fluorescence of *E. coli* OP50-GFP accumulated in the intestine of WT, *asic-1(ok415)*, *nhx-6(ok609)*, and *asic-1;nhx-6* worms at the indicated time points. AU, arbitrary units. p = 0.99911, 0.83781, 0.2944, 0.98598, 0.99146, 0.99859, 0.66288, 0.99158, 0.99999, 0.85303, 0.75236, 0.97564, 0.99999, 0.98559, 0.99999, 0.97419, 0.95167, and 0.99999. **d,** Colony-forming units (CFUs) of WT, *asic-1(ok415)*, *nhx-6(ok609)*, and *asic-1;nhx-6* worms exposed to *S. aureus* at the indicated time points , representing viable bacteria recovered from the intestine. CFUs quantify intestinal bacterial load, representing the number of viable bacteria recovered from the intestine following worm lysis and plating on selective

media. p = 0.97659, 0.84777, 0.99691, 1, 1, 1, 0.95623, 1, 0.89174, 0.06326, 0.25154, 0.32366, 0.01776, 0.00258, 0.0037, < 0.0001, 0.0001, and 0.0001. *$p < 0.05$, **$p < 0.01$, and ***$p < 0.001$ (one-way ANOVA with Tukey's post hoc test). Brackets contain the number of animals tested (**a,b**). *n* represents the number of animals tested (**c**) or independent assays (**d**). Data are shown as means ± SEM. Source data are provided as a Source Data file.

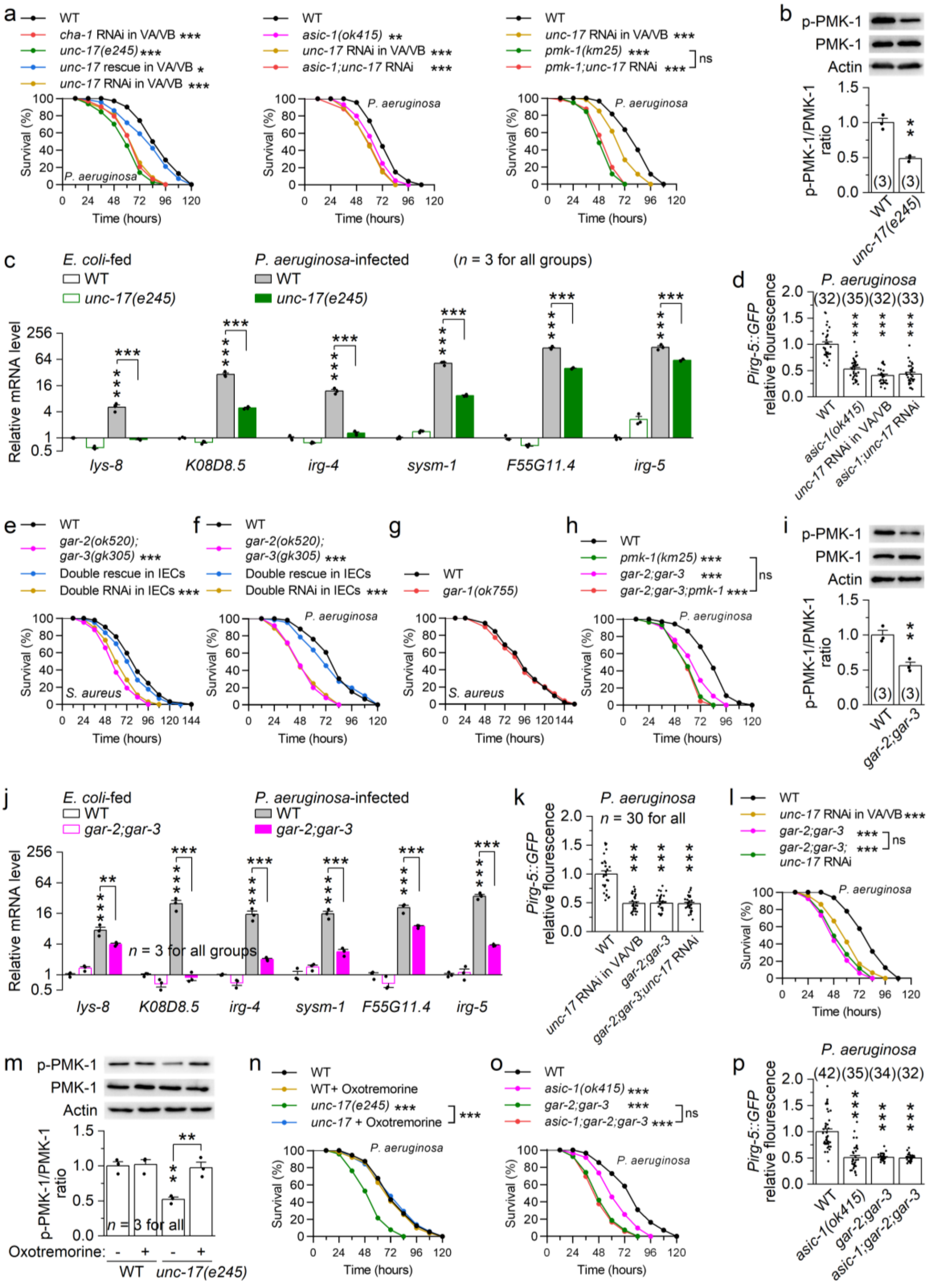

Supplementary Fig. 10 VA/VB-released ACh activates GAR-2;GAR-3 to promote PMK-1-dependent intestinal immunity during *P. aeruginosa* infection. a,e-h,l,o, Percent survival of the indicated genotypes on the full lawn of *P. aeruginosa* (**a,f,h,l,o**) or *S. aureus* (**e,g**). WT, wild type. RNAi of *cha-1* or *unc-17* was done in VA/VB using *Pdel-1*. *unc-17* rescue in VA/VB was done using *Pdel-1*. *gar-2;gar-3* double rescue or

double RNAi in IECs was done using *Pges-1*. p = < 0.0001, < 0.0001, 0.0759, < 0.0001, 0.0056, < 0.0001, < 0.0001, < 0.0001, < 0.0001, < 0.0001, and 0.2375 (**a**), < 0.0001, 0.0655, and < 0.0001 (**e**), < 0.0001, 0.6104, and < 0.0001 (**f**), 0.9584 (**g**), < 0.0001, < 0.0001, < 0.0001, and 0.5105 (**h**), < 0.0001, < 0.0001, < 0.0001, and 0.2297 (**l**), and < 0.0001, < 0.0001, < 0.0001, and 0.5315 (**o**). **b,i,** Representative immunoblots and quantification of the ratio of phosphorylated PMK-1 (p-PMK-1) to total PMK-1 in the indicated genotypes fed on *P. aeruginosa*. β-actin served as a loading control. p = 0.00131 (**b**) and 0.00626 (**i**). **c,j,** qRT-PCR analyses of the expression levels of six PMK-1/p38-dependent immune genes in the indicated genotypes fed on *E. coli* or *P. aeruginosa*. p = 0.8125, < 0.0001, < 0.0001, 0.99924, < 0.0001, < 0.0001, 0.99116, < 0.0001, < 0.0001, 0.99751, 0, < 0.0001, 0.99979, 0, < 0.0001, 0.99505, < 0.0001, and 0.0001 (**c**), and 0.96828, 0.0001, 0.00834, 0.99935, 0.0001, 0.0001, 0.99788, 0.0002, 0.0003, 0.98948, < 0.0001, < 0.0001, 0.99753, < 0.0001, 0.0006, 0.99995, < 0.0001, and < 0.0001 (**j**). **d,k,p,** Comparisons of *Pirg-5::GFP* relative fluorescence in the indicated genotypes fed on *P. aeruginosa*. p = 0, < 0.0001, 0.07013, < 0.0001 (**d**), 0, 0, 0.99989, 0 (**k**), and 0, 0, 0.99995, and 0 (**p**). **m,** Representative immunoblots and quantification of the ratio of p-PMK-1 to total PMK-1 in WT and *unc-17(e245)* worms fed on *P. aeruginosa* with or without 1 mM oxotremorine. p = 0.99507, 0.00282, 0.9909, and 0.00397. **n,** Percent survival of WT and *unc-17(e245)* worms on the full lawn of *P. aeruginosa* with or without 1 mM oxotremorine. p = 0.6587, < 0.0001, 0.8847, and < 0.0001. *$p < 0.05$, **$p < 0.01$, and ***$p < 0.001$ (log-rank (Kaplan-Meier) test for **a,e-h,l,n,o**; two-sided unpaired t test for **b,i**; one-way ANOVA with Tukey's post hoc test for **c,d,j,k,m,p**). ns, not significant. Brackets contain the number of animals tested (n). Data are shown as means ± SEM. Source data are provided as a Source Data file.

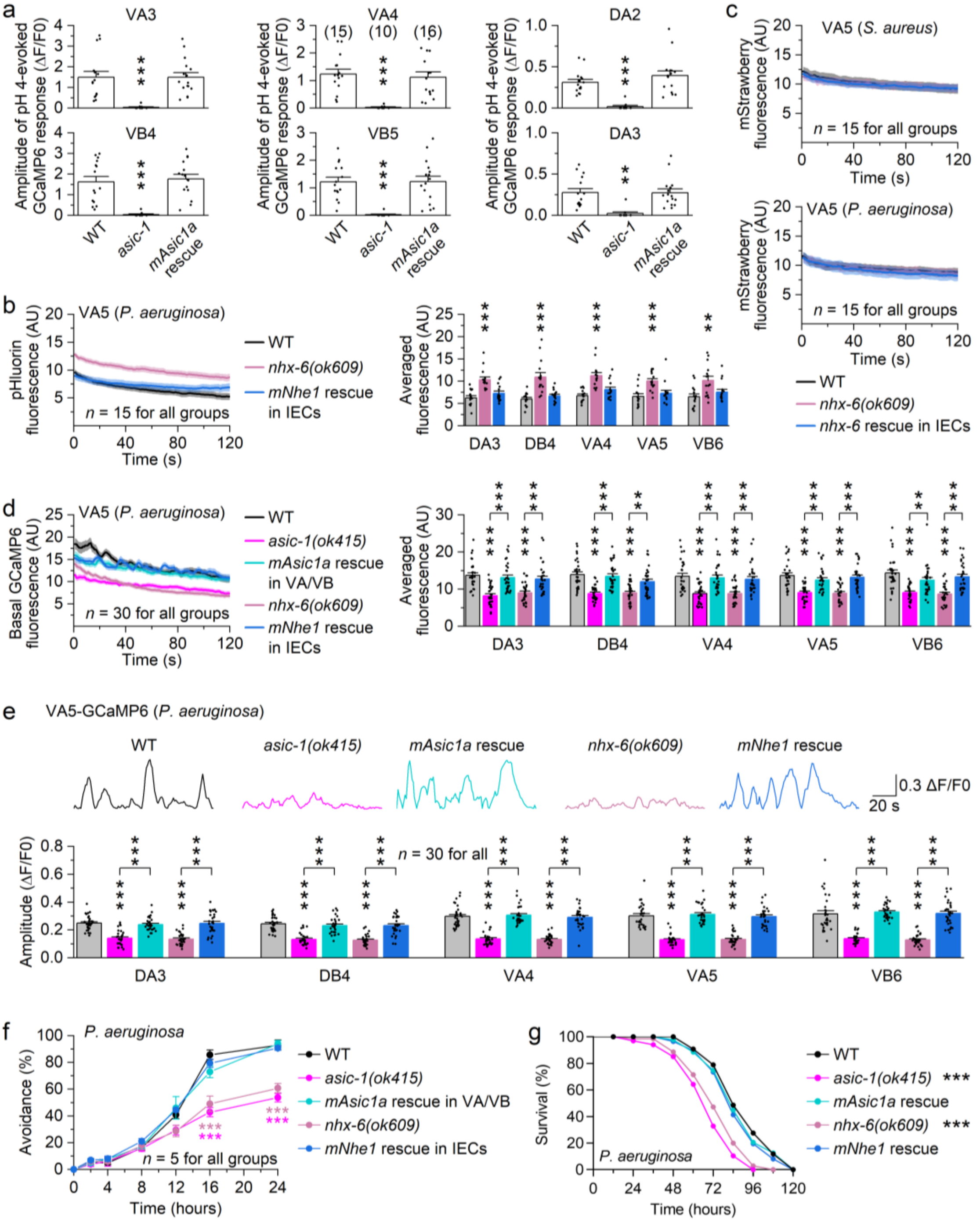

Supplementary Fig. 11 Mouse ASIC1a and NHE1 can functionally substitute for *C. elegans* ASIC-1 and NHX-6, respectively. a, Comparisons of pH 4-evoked GCaMP6 amplitude in motor neurons of wild type (WT), *asic-1(ok415)* mutants, and *mAsic1a* rescue worms. WT, wild type. Rescue was done by expressing mouse *Asic1a* (*mAsic1a*) in VA and VB using *Pdel-1*. p = 0.0005, 0.99978, 0.0001, 0.8664, 0.0003, 0.34817, < 0.0001, 0.87387, 0.0001, 0.99963, 0.00128, and 0.9998. **b,** pHluorin fluorescence in motor neurons of WT, *nhx-6(ok609)* mutants, and *mNhe1* rescue

worms fed on *P. aeruginosa*. Left, fluorescence of VA5 as representatives. Solid lines and shaded regions indicate the mean and SEM, respectively. Right, comparisons of averaged pHluorin fluorescence. AU, arbitrary units. Rescue was done by expressing mouse *Nhe1* (*mNhe1*) in IECs using *Pges-1*. p = < 0.0001, 0.3436, < 0.0001, 0.69623, < 0.0001, 0.16481, 0.0004, 0.66919, 0.00285, and 0.57557. **c,** mStrawberry fluorescence in motor neurons of pHluorin transgenic worms with the indicated genotypes fed on pathogenic bacteria. mStrawberry was co-expressed with pHluorin under *Punc-17Δ1* and is shown as a pH-insensitive expression control. Fluorescence of VA5 was shown as representatives. **d,** Basal GCaMP6 fluorescence in motor neurons of the indicated genotypes fed on *P. aeruginosa*. Left, fluorescence of VA5 as a representative. Right, comparisons of averaged basal fluorescence. p = < 0.0001, 0.95757, < 0.0001, < 0.0001, 0.78074, 0.0003, < 0.0001, 0.98117, < 0.0001, < 0.0001, 0.13204, 0.00226, < 0.0001, 0.99395, < 0.0001, < 0.0001, 0.90492, 0.0003, < 0.0001, 0.55357, 0.0003, < 0.0001, 0.97081, < 0.0001, < 0.0001, 0.19921, 0.00282, < 0.0001, 0.76581, and < 0.0001. **e,** Spontaneous GCaMP6 signals in motor neurons of the indicated genotypes fed on *P. aeruginosa*. Top, sample traces. Bottom, comparisons of the amplitude of spontaneous Ca^{2+} transients. p = < 0.0001, 0.9551, < 0.0001, 0, 0.99996, < 0.0001, 0, 0.86411, < 0.0001, 0, 0.87249, < 0.0001, 0, 0.97343, 0, 0, 0.99505, 0, 0, 0.95819, 0, 0, 0.99682, 0, 0, 0.96077, 0, 0, 0.99978, and 0. **f,** Pathogen avoidance of the indicated genotypes on the partial lawn of *P. aeruginosa*. p = 1, 1, 1, 0.66577, < 0.0001, < 0.0001, 1, 0.98977, 1, 0.99777, 0.35572, 1, 1, 1, 0.99977, 0.58561, < 0.0001, < 0.0001, 0.99818, 0.98528, 0.79471, 0.999911, 0.97124, and 0.99994. **g,** Percent survival of the indicated genotypes on the full lawn of *P. aeruginosa*. p = < 0.0001, 0.574, < 0.0001, and 0.2157. **$p < 0.01$ and ***$p < 0.001$ (one-way ANOVA with Tukey's post hoc test for **a,b,d,e**; two-way repeated-measures ANOVA with Sidak's multiple-comparisons correction for **f**; log-rank (Kaplan-Meier) test for **g**). Brackets contain the number of animals tested (**a**). *n* represents the number of animals tested (**b-e**) or independent assays (**f**). Data are shown as means ± SEM. Source data are provided as a Source Data file.

Supplementary Table 1. cDNA and RNAi primers used in this study.

Name of DNA fragment	PCR product length	**Sequence of primers** (F, forward; R, reverse)
asic-1 (cDNA)	2472 bp	F: ATAACCGGTATGGGAAAGAACAGCTTAAAACGG
		R: TGTGCCGGCTCAATTATCAAGATTAAACCCG
nhx-6 (cDNA)	1905 bp	F: ATGTCACTTATCCGTCGGCTG
		R: CTAAACCCGTTCTTCAAAATTG
mAsic1a (cDNA)	1581 bp	F: ATGGAACTGAAGACCGAGGAGGAG
		R: TTAGCAGGTAAAGTCCTCAAACG
mNhe1 (cDNA)	2463 bp	F: ATAACCGGTATGGGAAAGAACAGCTTAAAACGG
		R: TGTGCCGGCTCAATTATCAAGATTAAACCCG
gon-2 (cDNA)	6099 bp	F: ATGATGAGCGACGATATGCTTGACGAGAATGATG
		R: CTACACATCTGAACATTTTTGAGAGGTTAAATC
unc-17 (cDNA)	1599 bp	F: ATGGGCTTCAACGTGCCCGTC
		R: CTACCACTGCGGATTCAGTGGG
gar-2 (cDNA)	1845 bp	F: ATGGCGGTTGCAAGTGTGCTC
		R: TCAAACTCTTCGAAAATCTCC
gar-3 (cDNA)	1758 bp	F: ATGCAGTCCTCTTCGTTGGGGA
		R: CTAGTTGCGTCGGACATATCCC
gtl-1 RNAi	494 bp	F: ATGGACAGAAAACGTGGAAG
		R: GTATCTACTTCATGAATACCAGG
nhx-1 RNAi	470 bp	F: ATGAAGTCATTGGCTGTTATG
		R: TCGAATACTGATAAAACTGCC
nhx-2 RNAi	488 bp	F: TATGGTTGATTGGAGCATCG
		R: TCCGTCGTTCAATAGAGATTC
nhx-6 RNAi	488 bp	F: ATCGGGCTTCTGCTCATTTTG
		R: AGCAGCGGGGTTTCCACAGAG
cmd-1 RNAi	339 bp	F: ATGCGGTCTTTGGGACAAAATC
		R: CTTGGTTGTCATCATGGTGAC
cha-1 RNAi	479 bp	F: ATGGAAAAGGAAAAAGTTGACG
		R: TACTGTTCCATGCACATCTG
unc-17 RNAi	496 bp	F: GAATTGGGATGGCTCTTCGC
		R: CCATGAAGAGACGCCACATTG
aex-5 RNAi	530 bp	F: ATGAAATTAATTTTCCTGC
		R: TCAAACTCTTTAGAATTTTT
gar-2 RNAi	478 bp	F: ATGGCGGTTGCAAGTGTGCTCCTGG
		R: ATTCGGTTTCTTTCAGTATTCG
gar-3 RNAi	478 bp	F: ATGCAGTCCTCTTCGTTGGGGAATGCTG
		R: AAATGAGCAGAAGATTCAGGAC

Supplementary Table 2. qRT-PCR primers used in this study.

**Gene**	**Sequence of primers** (F, forward; R, reverse)
lys-8	F: TCAGTCTCCGTCAAGGTC
	R: GAAGCTGGCTCAATGAAA
K08D8.5	F: CCTGATGATAAGTATATT
	R: GGTTTTGGCTGTAGCACG
irg-4	F: TGGACAACCCAGATATGCAA
	R: GTATGCGATGGAAATGGACA
sysm-1	F: TGCTTCAGAGTCGTGTGTCG
	R: ACGCAGACACCACAGGTTTT
F55G11.4	F: GGATCCGTGTATTTGGCT
	R: GTGAAGACATATGTGCTC
irg-5	F: ACACAATCATTTGCGATGGA
	R: GGTAGTCATTGGAGCCGAAA
cwn-2	F: TGCTTCTTGGTGGTCAACAG
	R: CGTTGACATTCCTGAATAGC
ilys-2	F: TTTCTATTGCGGTCGCCTAC
	R: CGGTTGTAGTAGTTCTCAAC
ilys-3	F: TTCTGACTATTGCGGTCGCC
	R: CGGTTGTAGTAGTTCTCAAC
lys-5	F: GAATGCCAGAGCTGCTGGCC
	R: GCTAGCATGATCAGCTGGCC
cpr-2	F: ATGAACCTCATCCTTCTTTC
	R: ATTGAACTTCTCATGCATGC
clec-60	F: TGCGGCGATTCAAGCCGATC
	R: GCTCCGGATCCCTTGTAAGC

Uncropped scans of blots and gels.

Supplementary Fig. 8g

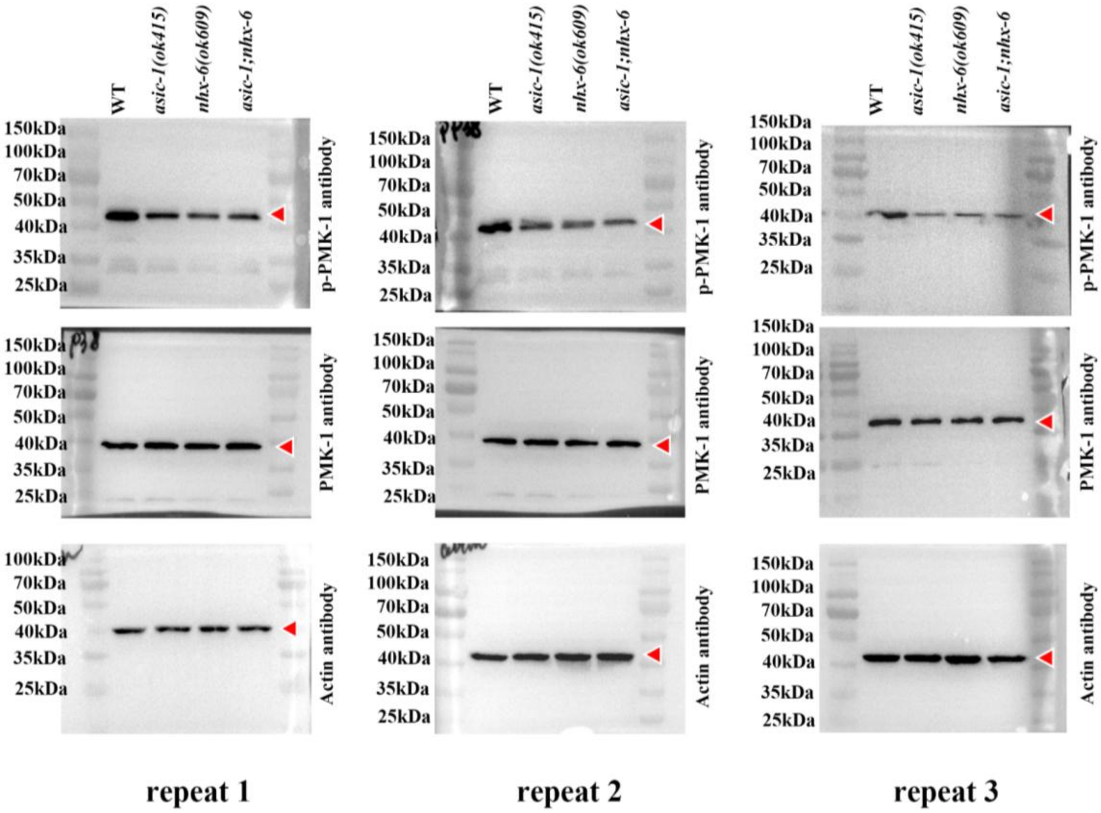

Supplementary Fig. 8h

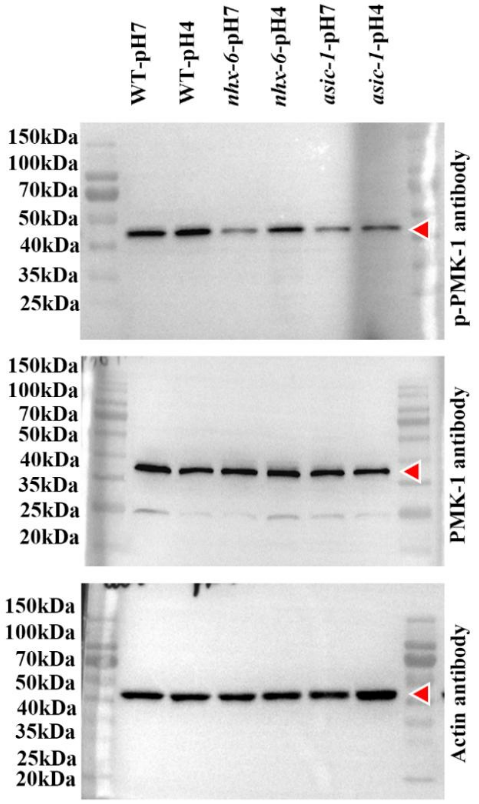

repeat 1

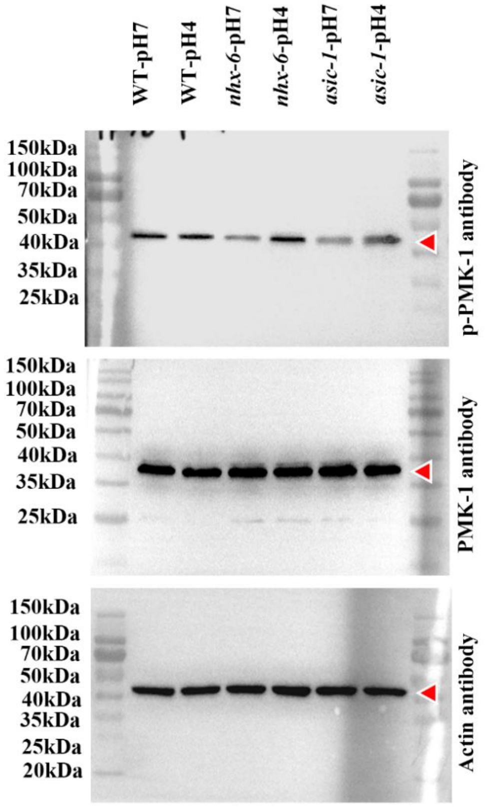

repeat 2

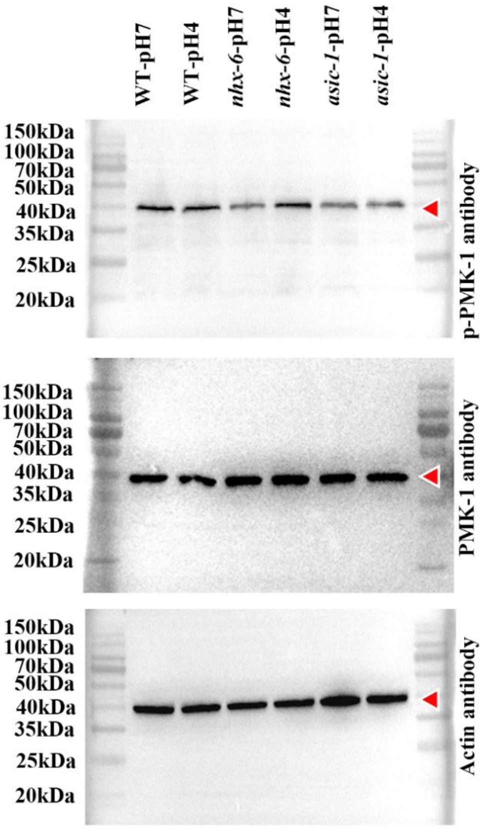

repeat 3

Supplementary Fig. 10b

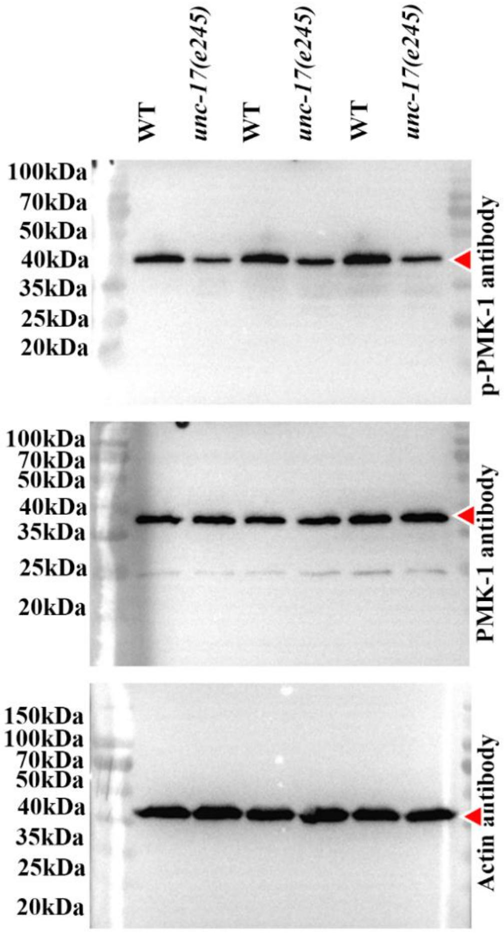

Supplementary Fig. 10i

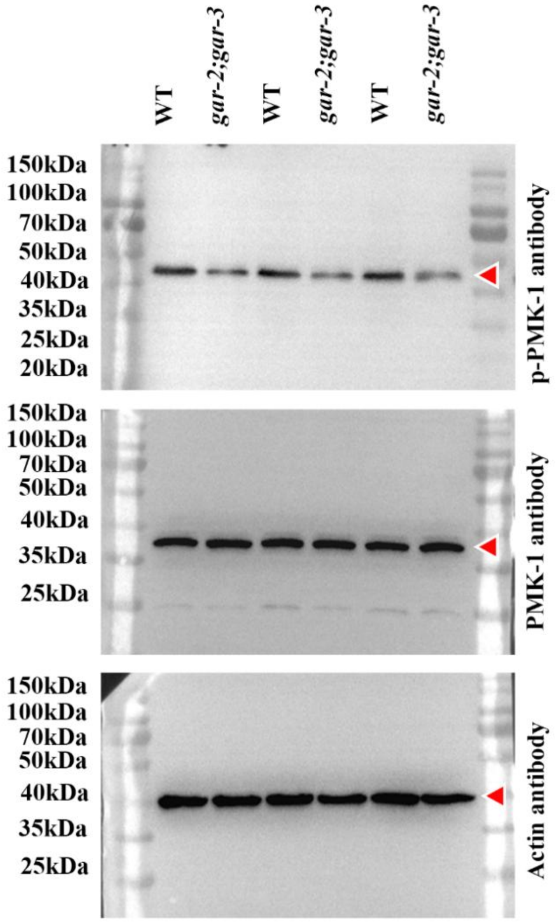

Supplementary Fig. 10m

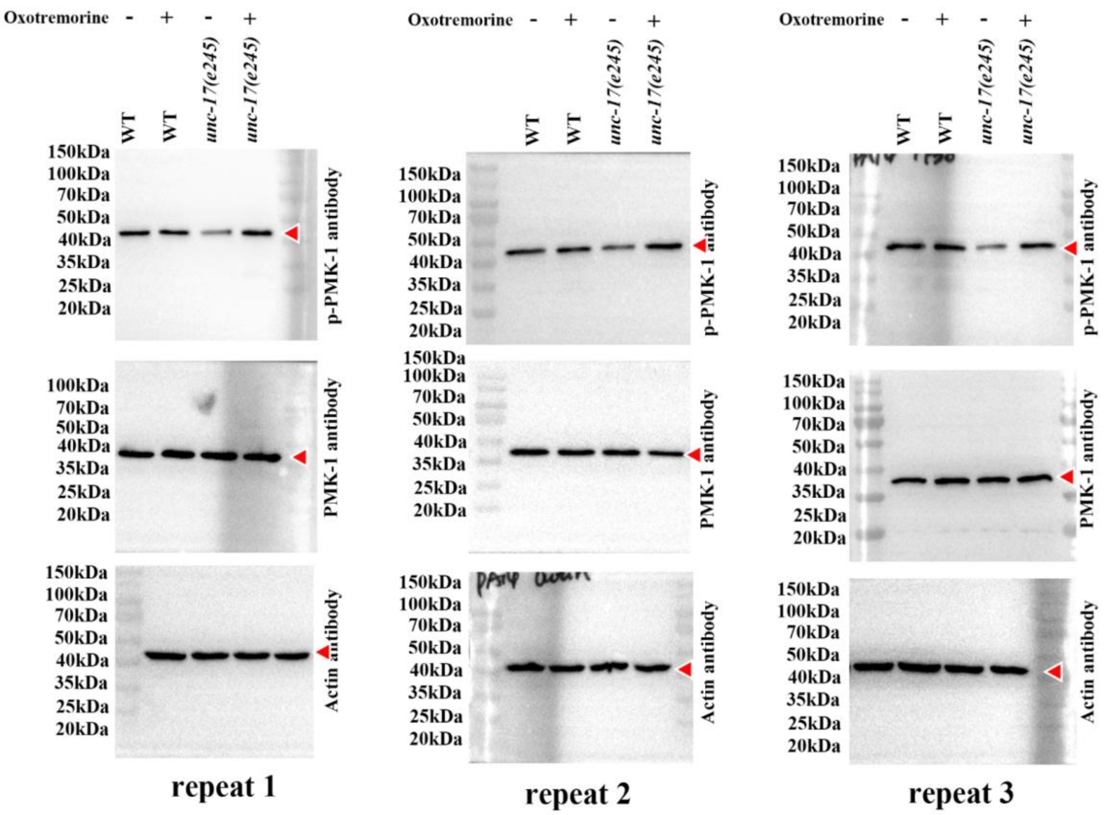

Supplement: Supplementary file 1 — Supplementary Information [file 41467_2026_71088_MOESM1_ESM.pdf]
