# Supplementary material for: Proton signaling links epithelial sensing to neural control of host defense in C. elegans
Source: Nat Commun. 2026 Mar 27;17:4493. doi: 10.1038/s41467-026-71088-6 (PMC13187020; doi:10.1038/s41467-026-71088-6)
Supplement: Supplementary file 2 — Description of Additional Supplementary Files [file 41467_2026_71088_MOESM2_ESM.pdf]

<u>Description of Additional Supplementary Files</u>

Supplementary Data 1: List of *C. elegans* strains
