# Supplementary material for: Proton signaling links epithelial sensing to neural control of host defense in C. elegans
Source: Nat Commun. 2026 Mar 27;17:4493. doi: 10.1038/s41467-026-71088-6 (PMC13187020; doi:10.1038/s41467-026-71088-6)
Supplement: Supplementary file 4 — Reporting Summary [file 41467_2026_71088_MOESM4_ESM.pdf]

nature portfolio

Corresponding author(s): Ping Liu

Last updated by author(s): Feb 24, 2026

Reporting Summary

Nature Portfolio wishes to improve the reproducibility of the work that we publish. This form provides structure for consistency and transparency in reporting. For further information on Nature Portfolio policies, see our Editorial Policies and the Editorial Policy Checklist.

Statistics

For all statistical analyses, confirm that the following items are present in the figure legend, table legend, main text, or Methods section.

n/a	Confirmed	
☐	☒	The exact sample size (*n*) for each experimental group/condition, given as a discrete number and unit of measurement
☐	☒	A statement on whether measurements were taken from distinct samples or whether the same sample was measured repeatedly
☐	☒	The statistical test(s) used AND whether they are one- or two-sided<br>*Only common tests should be described solely by name; describe more complex techniques in the Methods section.*
☒	☐	A description of all covariates tested
☒	☐	A description of any assumptions or corrections, such as tests of normality and adjustment for multiple comparisons
☐	☒	A full description of the statistical parameters including central tendency (e.g. means) or other basic estimates (e.g. regression coefficient) AND variation (e.g. standard deviation) or associated estimates of uncertainty (e.g. confidence intervals)
☐	☒	For null hypothesis testing, the test statistic (e.g. *F*, *t*, *r*) with confidence intervals, effect sizes, degrees of freedom and *P* value noted<br>*Give P values as exact values whenever suitable.*
☒	☐	For Bayesian analysis, information on the choice of priors and Markov chain Monte Carlo settings
☒	☐	For hierarchical and complex designs, identification of the appropriate level for tests and full reporting of outcomes
☒	☐	Estimates of effect sizes (e.g. Cohen's *d*, Pearson's *r*), indicating how they were calculated

Our web collection on statistics for biologists contains articles on many of the points above.

Software and code

Policy information about availability of computer code

Data collection	MShot Image Analysis System (version 1.1) was used for imaging collection; Track-A-Worm (version 2.0) was used for locomotion tests and was previously published in Wang, S. J. and Wang, Z. W., 2013; Whole-cell current recordings were performed using Clampex 10 (Molecular Devices).
Data analysis	Calcium imaging data were analyzed using ImageJ (version 1.50i); Locomotion behavior was analyzed using Track-A-Worm (version 2.0); ; Whole-cell current data were analyzed using Clampfit 10 (Molecular Devices); Statistical analyses and data graphing were performed using Origin 2019 (OriginLab), Prism 8 (GraphPad), or IBM SPSS Statistics 21 (IBM).

For manuscripts utilizing custom algorithms or software that are central to the research but not yet described in published literature, software must be made available to editors and reviewers. We strongly encourage code deposition in a community repository (e.g. GitHub). See the Nature Portfolio guidelines for submitting code & software for further information.

Data

Policy information about availability of data

All manuscripts must include a data availability statement. This statement should provide the following information, where applicable:

- Accession codes, unique identifiers, or web links for publicly available datasets
- A description of any restrictions on data availability
- For clinical datasets or third party data, please ensure that the statement adheres to our policy

All data generated or analyzed during this study are included in this published article and its supplementary information files. Source data are provided with this

paper.

Research involving human participants, their data, or biological material

Policy information about studies with human participants or human data. See also policy information about sex, gender (identity/presentation), and sexual orientation and race, ethnicity and racism.

Reporting on sex and gender	All experiments were performed using young adult hermaphrodites.
Reporting on race, ethnicity, or other socially relevant groupings	not applicable
Population characteristics	not applicable
Recruitment	not applicable
Ethics oversight	The study uses the nematode C. elegans, which does not require Ethical approval.

Note that full information on the approval of the study protocol must also be provided in the manuscript.

Field-specific reporting

Please select the one below that is the best fit for your research. If you are not sure, read the appropriate sections before making your selection.

[x] Life sciences   [ ] Behavioural & social sciences   [ ] Ecological, evolutionary & environmental sciences

For a reference copy of the document with all sections, see nature.com/documents/nr-reporting-summary-flat.pdf

Life sciences study design

All studies must disclose on these points even when the disclosure is negative.

Sample size	Sample sizes were determined based on previous studies using similar experimental paradigms, including our previous work. No statistical methods were used to predetermine sample sizes. Instead, sample sizes were chosen to ensure adequate power to detect biological differences, and are consistent with those commonly used in the field.
Data exclusions	No data were excluded from the analyses.
Replication	Replication was achieved using different cells, different animals, or independent assays. All experiments were performed on at least two separate days. For imaging, locomotion, and whole-cell current analyses, n represents the number of animals tested, whereas for behavioral avoidance assays, n denotes the number of independent assays. qRT-PCR and western blot experiments were independently repeated three times. We are not aware of any results that could not be reproduced under the described experimental conditions.
Randomization	For each genotype, animals grown on the same plate were randomly allocated to different conditions. For individual assays, animals were randomly selected. For population assays, animals from a plate were washed off with M9 buffer, and adequate amount of buffer containing animals was placed onto each choice plate.
Blinding	Blinding was not applied. Locomotion tests were conducted using the automated Track-A-Worm system, minimizing researcher involvement. For other assays, most animals were readily identifiable based on transgenic markers, visible phenotypes, or bacterial treatments.

Reporting for specific materials, systems and methods

We require information from authors about some types of materials, experimental systems and methods used in many studies. Here, indicate whether each material, system or method listed is relevant to your study. If you are not sure if a list item applies to your research, read the appropriate section before selecting a response.

Materials & experimental systems

n/a	Involved in the study	
[ ]	[x]	Antibodies
[x]	[ ]	Eukaryotic cell lines
[x]	[ ]	Palaeontology and archaeology
[ ]	[x]	Animals and other organisms
[x]	[ ]	Clinical data
[x]	[ ]	Dual use research of concern
[x]	[ ]	Plants

Methods

n/a	Involved in the study	
[x]	[ ]	ChIP-seq
[x]	[ ]	Flow cytometry
[x]	[ ]	MRI-based neuroimaging

Antibodies

Antibodies used	rabbit anti-phospho-p38 MAPK monoclonal antibody (1:1000; ABclonal, AP0526), rabbit anti-p38 MAPK monoclonal antibody (1:1000; Abcam, ab170099), and mouse anti-β-actin monoclonal antibody (1:5000; ABclonal, AC004).
Validation	rabbit anti-phospho-p38 MAPK monoclonal antibody (ABclonal, AP0526), https://abclonal.com.cn/catalog/AP0526;<br>rabbit anti-p38 MAPK monoclonal antibody (Abcam, ab170099), https://www.abcam.com/en-us/products/primary-antibodies/p38-alpha-mapk14-antibody-e229-ab170099;<br>mouse anti-β-actin monoclonal antibody (ABclonal, AC004), https://abclonal.com.cn/catalog/AC004.

Animals and other research organisms

Policy information about studies involving animals; ARRIVE guidelines recommended for reporting animal research, and Sex and Gender in Research

Laboratory animals

The following C. elegans strains were used:
wild type CGC N2(Bristol)

RB680	CGC	asic-1(ok415)
VC383	CGC	nhx-6(ok609)
PLX529	This paper	asic-1(ok415);nhx-6(ok609)
EJ26	Jianke Gong	gon-2(q362)
PLX1045	This paper	asic-1(ok415);gon-2(q362)
VC636	CGC	cwn-2(ok895)
PLX870	This paper	asic-1(ok415);cwn-2(ok895)
CB933	CGC	unc-17(e245)
PLX187	This paper	gar-2(ok520);gar-3(gk305)
PLX186	This paper	asic-1(ok415);gar-2(ok520);gar-3(gk305)
KU25	CGC	pmk-1(km25)
PLX877	This paper	asic-1(ok415);pmk-1(km25)
RB896	CGC	gar-1(ok755)
PLX880	This paper	gar-2(ok520);gar-3(gk305);pmk-1(km25)
PLX156	This paper	xyhIs156[Punc-17Δ1::GCaMP6s]
PLX37	This paper	unc-13(e51);xyhIs156[Punc-17Δ1::GCaMP6s]
PLX38	This paper	unc-31(e169);xyhIs156[Punc-17Δ1::GCaMP6s]
PLX158	This paper	asic-1(ok415);xyhIs156[Punc-17Δ1::GCaMP6s]
PLX777	This paper	xyhIs156[Punc-17Δ1::GCaMP6s];xyhEx777[Pdel-1::SL2::TeTx::mCherry, Pmyo-2::mStrawberry]
PLX164	This paper	asic-1(ok415);xyhIs156[Punc-17Δ1::GCaMP6s];xyhEx164[Pdel-1::SL2::asic-1(cDNA)::SL2::mStrawberry, Pmyo-2::mStrawberry]
PLX469	This paper	xyhIs469[Punc-17Δ1::pHluorin::Linker::hCD8::mStrawberry, Pmyo-2::mStrawberry]
PLX311	This paper	nhx-6(ok609);xyhIs469[Punc-17Δ1::pHluorin::Linker::hCD8::mStrawberry, Pmyo-2::mStrawberry]
PLX694	This paper	xyhIs694[Pges-1::GCaMP6s, lin-15(+)]
PLX893	This paper	trpa-1(ok999);xyhIs694[Pges-1::GCaMP6s, lin-15(+)]
PLX933	This paper	xyhIs694[Pges-1::GCaMP6s, lin-15(+)];xyhEx933[Pges-1::gtl-1 RNAi, Pmyo-2::mStrawberry]
PLX905	This paper	gon-2(q362);xyhIs694[Pges-1::GCaMP6s, lin-15(+)]
PLX946	This paper	xyhEx946[Pges-1::gon-2(cDNA)::SL2::mCherry, Pmyo-2::mStrawberry];gon-2(q362);xyhIs694[Pges-1::GCaMP6s, lin-15(+)]
PLX908	This paper	gon-2(q362);xyhIs469[Punc-17Δ1::pHluorin::Linker::hCD8::mStrawberry, Pmyo-2::mStrawberry]
PLX1169	This paper	gon-2(q362);xyhIs469[Punc-17Δ1::pHluorin::Linker::hCD8::mStrawberry, Pmyo-2::mStrawberry];xyhEx1169 [Pges-1::gon-2(cDNA), Pmyo-2::GFP]
PLX1044	This paper	nhx-6(ok609);gon-2(q362);xyhIs469[Punc-17Δ1::pHluorin::Linker::hCD8::mStrawberry, Pmyo-2::mStrawberry]
PLX911	This paper	gon-2(q362);xyhIs156[Punc-17Δ1::GCaMP6s]
PLX1163	This paper	gon-2(q362);xyhIs156[Punc-17Δ1::GCaMP6s];xyhEx1163[Pges-1::gon-2(cDNA), Pmyo-2::mStrawberry]
PLX1162	This paper	nhx-6(ok609);gon-2(q362);xyhIs156[Punc-17Δ1::GCaMP6s]
PLX775	This paper	nhx-6(ok609);xyhEx763[Pges-1::cmd-1 RNAi, Pmyo-2::GFP];xyhIs469 [Punc-17Δ1::PHluorin::Linker::hCD8::mStrawberry, Pmyo-2::mStrawberry]
PLX763	This paper	xyhEx763[Pges-1::cmd-1 RNAi, Pmyo-2::GFP];xyhIs469[Punc-17Δ1::PHluorin::Linker::hCD8::mStrawberry, Pmyo-2::mStrawberry]
PLX614	This paper	nhx-6(ok609);xyhIs156[Punc-17Δ1::GCaMP6s]
PLX793	This paper	nhx-6(ok609);xyhIs156[Punc-17Δ1::GCaMP6s];xyhEx593[Pges-1::nhx-6(cDNA)::SL2::mCherry, Pmyo-2::mStrawberry]
PLX602	This paper	asic-1(ok415);nhx-6;xyhIs156[Punc-17Δ1::GCaMP6s]
PLX244	This paper	asic-1(ok415);xyhEx244[Punc-17Δ1::asic-1(cDNA)::SL2::mStrawberry, Pmyo-2::mStrawberry]
PLX591	This paper	nhx-6(ok609);xyhEx591[Pges-1::nhx-6(cDNA)::SL2::mCherry, Pmyo-2::mStrawberry]
PLX690	This paper	xyhEx690[Pges-1::cwn-2 RNAi, Pmyo-2::mStrawberry]
PLX1057	This paper	glo-4(ok623);xyhEx1051[Pnhx-6::nhx-6 cDNA::mStrawberry, Pasic-1::asic-1::GFP]
PLX861	This paper	cwn-2(ok895);xyhEx861[Pges-1::cwn-2(cDNA)::SL2::mCherry, Pmyo-2::mStrawberry]
JIN810	CGC	agIs26[Pclec-60::GFP, Pmyo-2::mCherry]
PLX594	This paper	asic-1(ok415);agIs26[Pclec-60::GFP, Pmyo-2::mCherry]

PLX731	This paper	nhx-6(ok609);agIs26[Pclec-60::GFP, Pmyo-2::mCherry]
PLX732	This paper	asic-1(ok415);nhx-6(ok609);agIs26[Pclec-60::GFP, Pmyo-2::mCherry]
PLX792	This paper	asic-1(ok415);agIs26[Pclec-60::GFP, Pmyo-2::mCherry];xyhEx603[Pdel-1::SL2::asic-1(cDNA)::SL2::mStrawberry, Pmyo-2::mStrawberry]
PLX789	This paper	nhx-6(ok609);agIs26[Pclec-60::GFP, Pmyo-2::mCherry];xyhEx591[Pges-1::nhx-6(cDNA)::SL2::mCherry, Pmyo-2::mStrawberry]
PLX629	This paper	xyhEx629[Pdel-1::SL2::cha-1 RNAi, Pmyo-2::GFP]
PLX917	This paper	unc-17(e245);xyhEx917[Pdel-1::SL2::unc-17(cDNA), Pmyo-2::mStrawberry]
PLX687	This paper	xyhEx687[Pdel-1::SL2::unc-17 RNAi, Pmyo-2::mStrawberry]
PLX728	This paper	asic-1(ok415);xyhEx687[Pdel-1::SL2::unc-17 RNAi, Pmyo-2::mStrawberry]
PLX1046	This paper	cwn-2(ok895);xyhEx687[Pdel-1::SL2::unc-17 RNAi, Pmyo-2::mStrawberry]
PLX746	This paper	xyhEx687[Pdel-1::SL2::unc-17 RNAi, Pmyo-2::mStrawberry];agIs26[Pclec-60::GFP, Pmyo-2::mCherry]
PLX747	This paper	asic-1(ok415);xyhEx687[Pdel-1::SL2::unc-17 RNAi, Pmyo-2::mStrawberry];agIs26[Pclec-60::GFP, Pmyo-2::mCherry]
PLX926	This paper	gar-2(ok520)III;gar-3(gk305);agIs26[Pclec-60::GFP, Pmyo-2::mCherry]
PLX1047	This paper	asic-1(ok415);gar-2(ok520)III;gar-3(gk305);agIs26[Pclec-60::GFP, Pmyo-2::mCherry]
PLX715	This paper	asic-1(ok415);xyhIs156[Punc-17Δ1::GCaMP6s];xyhEx606[Pdel-1::SL2::mASIC1a(cDNA)::SL2::mStrawberry, Punc-122::dsRed]
PLX794	This paper	nhx-6(ok609);xyhIs156[Punc-17Δ1::GCaMP6s];xyhEx794[Pges-1::mNhe1(cDNA), Pmyo-2::mStrawberry]
PLX657	This paper	nhx-6(ok609);xyhIs469[Punc-17Δ1::pHluorin::Linker::hCD8::mStrawberry, Pmyo-2::mStrawberry];xyhEx662 [Pges-1::mNHE1(cDNA), Pmyo-2::GFP]
PLX964	This paper	unc-9(fc16);xyhIs156[Punc-17Δ1::GCaMP6s];xyhEx964[Punc-17Δ1::inx-3 RNAi, Punc-17Δ1::inx-12 RNAi, Pmyo-2::mStrawberry]
PLX967	This paper	unc-9(fc16);inx-7(tm2738);xyhIs156[Punc-17Δ1::GCaMP6s];xyhEx967[Punc-17Δ1::inx-3 RNAi, Pmyo-2::mStrawberry]
PLX684	This paper	xyhIs469[Punc-17Δ1::pHluorin::Linker::hCD8::mStrawberry, Pmyo-2::mStrawberry];xyhEx684[Pges-1::nhx-1 RNAi, Pmyo-2::GFP]
PLX668	This paper	xyhIs469[Punc-17Δ1::pHluorin::Linker::hCD8::mStrawberry, Pmyo-2::mStrawberry]; xyhEx668[Pges-1::nhx-2 RNAi, Pmyo-2::GFP]
PLX656	This paper	nhx-3(ok1049) V;xyhIs469[Punc-17Δ1::pHluorin::Linker::hCD8::mStrawberry, Pmyo-2::mStrawberry]
PLX667	This paper	nhx-4(ok668);xyhIs469[Punc-17Δ1::pHluorin::Linker::hCD8::mStrawberry, Pmyo-2::mStrawberry]
PLX649	This paper	nhx-5(ok661); xyhIs469[Punc-17Δ1::pHluorin::Linker::hCD8::mStrawberry, Pmyo-2::mStrawberry]
PLX633	This paper	nhx-7(ok583);xyhIs469[Punc-17Δ1::pHluorin::Linker::hCD8::mStrawberry, Pmyo-2::mStrawberry]
PLX650	This paper	nhx-8(ok549); xyhIs469[Punc-17Δ1::pHluorin::Linker::hCD8::mStrawberry, Pmyo-2::mStrawberry]
PLX652	This paper	nhx-9(ok847);xyhIs469[Punc-17Δ1::pHluorin::Linker::hCD8::mStrawberry, Pmyo-2::mStrawberry]
PLX616	This paper	asic-1(ok415);xyhIs156[Punc-17Δ1::GCaMP6s];xyhEx616[Pmyo-3::HisCl::SL2::mStrawberry, Pmyo-2::mStrawberry]
PLX776	This paper	del-1(ok150);xyhIs156[Punc-17Δ1::GCaMP6s];xyhEx616[Pmyo-3::HisCl::SL2::mStrawberry, Pmyo-2::mStrawberry]
PLX661	This paper	asic-1(ok415);xyhEx625[Pdel-1::SL2::TRPV1::SL2::GCaMP6s, Punc-122::dsRed]
PLX241	This paper	asic-1(ok415);xyhEx241[Pdat-1::asic-1(cDNA)::SL2::mStrawberry, Pmyo-2::mStrawberry]
PLX723	This paper	xyhEx723[Pges-1::pmk-1 RNAi, Pmyo-2::mStrawberry]
PLX859	This paper	pmk-1(km25);xyhEx859[Pges-1::pmk-1(cDNA)::SL2::mCherry, Pmyo-2::mStrawberry]
AY101	CGC	acIs101[Pirg-5::GFP, rol-6(su1006)]
PLX521	This paper	asic-1(ok415);acIs101[Pirg-5::GFP, rol-6(su1006)]
PLX696	This paper	asic-1(ok415);acIs101[Pirg-5::GFP, rol-6(su1006)];xyhEx603[Pdel-1::SL2::asic-1(cDNA)::SL2::mStrawberry, Pmyo-2::mStrawberry]
PLX697	This paper	nhx-6(ok609);acIs101[Pirg-5::GFP, rol-6(su1006)]
PLX791	This paper	nhx-6(ok609);acIs101[Pirg-5::GFP, rol-6(su1006)];xyhEx591[Pges-1::nhx-6(cDNA)::SL2::mCherry, Pmyo-2::mStrawberry]
PLX698	This paper	asic-1(ok415);nhx-6(ok609);acIs101[Pirg-5::GFP, rol-6(su1006)]
PLX756	This paper	xyhEx687[Pdel-1::SL2::unc-17 RNAi, Pmyo-2::mStrawberry];acIs101[Pirg-5::GFP, rol-6(su1006)]
PLX757	This paper	asic-1(ok415);xyhEx687[Pdel-1::SL2::unc-17 RNAi, Pmyo-2::mStrawberry];acIs101[Pirg-5::GFP, rol-6(su1006)]
PLX863	This paper	gar-2(ok520);gar-3(gk305);xyhEx863[Pges-1::gar-2(cDNA)::SL2::mCherry, Pges-1::gar-3(cDNA)::SL2::mCherry, Pmyo-2::mStrawberry]
PLX252	This paper	xyhEx252[Pges-1::gar-2 RNAi, Pges-1::gar-3 RNAi, Pmyo-2::GFP]
PLX978	This paper	gar-2(ok520);gar-3(gk305);acIs101[Pirg-5::GFP, rol-6(su1006)]
PLX1048	This paper	gar-2(ok520)III;gar-3(gk305);xyhEx687[Pdel-1::SL2::unc-17 RNAi, Pmyo-2::mStrawberry];acIs101[Pirg-5::GFP, rol-6(su1006)]

Wild animals

The study did not involve wild animals.

Reporting on sex

All experiments were conducted using young adult hermaphrodites.

Field-collected samples

The study did not involve samples collected from the field.

Ethics oversight

The study uses the nematode C. elegans, which does not require ethical approval.

Note that full information on the approval of the study protocol must also be provided in the manuscript.

Plants

Seed stocks	not applicable
Novel plant genotypes	not applicable
Authentication	not applicable
